# Immediate and long-term electrophysiological biomarkers of antidepressant-like behavioral effects after subanesthetic ketamine and medial prefrontal cortex deep brain stimulation treatment

**DOI:** 10.3389/fnins.2024.1389096

**Published:** 2024-06-20

**Authors:** Matthew Bergosh, Sasha Medvidovic, Nancy Zepeda, Lindsey Crown, Jennifer Ipe, Lauren Debattista, Luis Romero, Eimon Amjadi, Tian Lam, Erik Hakopian, Wooseong Choi, Kevin Wu, Jack Yu Tung Lo, Darrin Jason Lee

**Affiliations:** ^1^Department of Neurological Surgery, Keck School of Medicine, University of Southern California, Los Angeles, CA, United States; ^2^Neurorestoration Center, Keck School of Medicine, University of Southern California, Los Angeles, CA, United States; ^3^Department of Ophthalmology, Keck School of Medicine, University of Southern California, Los Angeles, CA, United States; ^4^Department of Psychiatry and Behavioral Sciences, Keck School of Medicine, University of Southern California, Los Angeles, CA, United States; ^5^Department of Bioengineering, University of California Riverside, Riverside, CA, United States; ^6^Rancho Los Amigos National Rehabilitation Center, Downey, CA, United States

**Keywords:** electrophysiology, biomarker, depression, behavior, deep brain stimulation, ketamine, psychedelic, translational

## Abstract

**Introduction:**

Both ketamine (KET) and medial prefrontal cortex (mPFC) deep brain stimulation (DBS) are emerging therapies for treatment-resistant depression, yet our understanding of their electrophysiological mechanisms and biomarkers is incomplete. This study investigates aperiodic and periodic spectral parameters, and the signal complexity measure sample entropy, within mPFC local field potentials (LFP) in a chronic corticosterone (CORT) depression model after ketamine and/or mPFC DBS.

**Methods:**

Male rats were intraperitoneally administered CORT or vehicle for 21 days. Over the last 7 days, animals receiving CORT were treated with mPFC DBS, KET, both, or neither; then tested across an array of behavioral tasks for 9 days.

**Results:**

We found that the depression-like behavioral and weight effects of CORT correlated with a decrease in aperiodic-adjusted theta power (5–10 Hz) and an increase in sample entropy during the administration phase, and an increase in theta peak frequency and a decrease in the aperiodic exponent once the depression-like phenotype had been induced. The remission-like behavioral effects of ketamine alone correlated with a post-treatment increase in the offset and exponent, and decrease in sample entropy, both immediately and up to eight days post-treatment. The remission-like behavioral effects of mPFC DBS alone correlated with an immediate decrease in sample entropy, an immediate and sustained increase in low gamma (20–50 Hz) peak width and aperiodic offset, and sustained improvements in cognitive function. Failure to fully induce remission-like behavior in the combinatorial treatment group correlated with a failure to suppress an increase in sample entropy immediately after treatment.

**Conclusion:**

Our findings therefore support the potential of periodic theta parameters as biomarkers of depression-severity; and periodic low gamma parameters and cognitive measures as biomarkers of mPFC DBS treatment efficacy. They also support sample entropy and the aperiodic spectral parameters as potential cross-modal biomarkers of depression severity and the therapeutic efficacy of mPFC DBS and/or ketamine. Study of these biomarkers is important as objective measures of disease severity and predictive measures of therapeutic efficacy can be used to personalize care and promote the translatability of research across studies, modalities, and species.

## 1 Introduction

Depression is the most common psychiatric condition globally, affecting an estimated 246 million people in 2020 (Santomauro et al., 2021), with an increasing prevalence in recent years (Goodwin et al., 2022). Importantly, current medications are only effective for 30–40% of patients and require weeks to achieve a therapeutic effect. This leaves an ever-increasing unmet need for more effective treatments (Blackburn, 2019). Mounting preclinical and clinical evidence has increased interest in emerging treatments such as deep brain stimulation (DBS) of the medial prefrontal cortex (mPFC) (Dandekar et al., 2018), as well as subanesthetic doses of ketamine (Bobo et al., 2016). However, how these treatment modalities achieve their therapeutic efficacy is not fully understood.

Oscillatory, or periodic, activity in local field potential (LFP) and electroencephalogram (EEG) recordings has been used to study the neurophysiological effects of both treatment modalities in depression. Nearly every canonical frequency band has been implicated as a potential biomarker or mechanism of depression or remission, especially within the mPFC (Sun et al., 2015; Fitzgerald and Watson, 2018; Jia et al., 2019). However, the majority of previous studies ignored or removed broadband aperiodic changes in the power spectra, potentially confounding the analysis of narrowband periodic activity, as well as missing important physiological information. Therefore, techniques have been developed that algorithmically separate the aperiodic, broadband component from narrowband, periodic peak components. The aperiodic component is parameterized into an exponential function with an exponent describing its steepness and an offset describing its vertical shift, while the periodic peaks over and above this aperiodic component are described by Gaussian curves possessing amplitude, width, and center frequency parameters (Donoghue et al., 2020). Changes in these aperiodic and aperiodic-adjusted periodic parameters (together known as spectral parameters) in the mPFC due to depression and treatment have been explored in task-based (Stolz et al., 2023) and resting-state paradigms (Huang et al., 2021) in humans, as well as *in-silico* through cortical microcircuit models of depression (Mazza et al., 2023) and novel antidepressants (Guet-McCreight et al., 2024). Besides spectral parameters, sample entropy, a measure of signal irregularity and complexity, has been studied as a potential biomarker and clue regarding the neural changes that underlie depression in humans (Faust et al., 2014; Acharya et al., 2015; Čukić et al., 2020; Lin et al., 2020), as it has been shown to represent the functional activity, processing, and connectivity of a region (Wang et al., 2018). However, less work has been done after treatment (Maltbie et al., 2020), or in reverse translating these findings to rodents (Zheng et al., 2012). Therefore, this study will be among the first to explore the translatability and utility of both spectral parameterization and sample entropy in rodent models of depression and treatment.

Furthermore, despite a wealth of publications investigating ketamine and DBS independently, few studies have directly compared the two within the same study (Willner et al., 2019). Such a comparison can help control for differing methodologies in behavioral assays, electrophysiological recording procedures, and analysis techniques. Additionally, no studies have attempted to combine these treatment modalities, despite evidence of overlapping mechanisms, such as beta and gamma power modulation (Bambico et al., 2015; Jett et al., 2015; Sun et al., 2015; Sumner et al., 2020). Furthermore, few preclinical studies have connected long-term changes (>24 h) in mPFC activity after DBS or ketamine treatment with the immediate changes in the region, or the sustained antidepressant-like behavioral effects. In the present study, we investigate electrophysiological changes in the rat mPFC (prelimbic cortex), and behavioral effects of mPFC DBS and/or ketamine administration in a chronic corticosterone (CORT) preclinical model of depression (Sterner and Kalynchuk, 2010). We hypothesized that a combination of ketamine and DBS would induce synergistic behavioral and electrophysiological antidepressant-like effects. LFP recordings immediately, one day, and eight days after treatment administration were compared to baseline recordings and between groups, and then correlated with depression- or remission-like performance in a behavioral assay. We hypothesized that changes in the activity of the mPFC would parallel the depression-like action of CORT and the antidepressant-like action of ketamine and mPFC DBS, and could therefore act as biomarkers.

## 2 Methods

### 2.1 Animal numbers, housing, and groups

42 male 6–8 week old (350 ± 50 g) Sprague Dawley rats (Charles River Laboratories, Wilmington, MA) were obtained. The animals acclimated to the facilities for 1 week before experimental procedures began. Animals had access to food and water *ad libitum*, except for 24 h preceding Sucrose Preference Tests (SPT). The animals were single-housed in a controlled environment at 23 ± 1°C, 55–65% relative humidity, and a fixed 12 h light/dark cycle with lights on at 0600. All procedures performed were approved by the University of Southern California Institutional Animal Care and Use Committee. Animals were weighed and observed daily for the duration of the experiment. The animals were randomly placed into one of five groups: control (*n* = 8), CORT (*n* = 8), CORT+KET (*n* = 9), CORT+DBS (*n* = 8), and CORT+DBS+KET (*n* = 9).

### 2.2 Electrode implantation surgery

**Figure 1:**
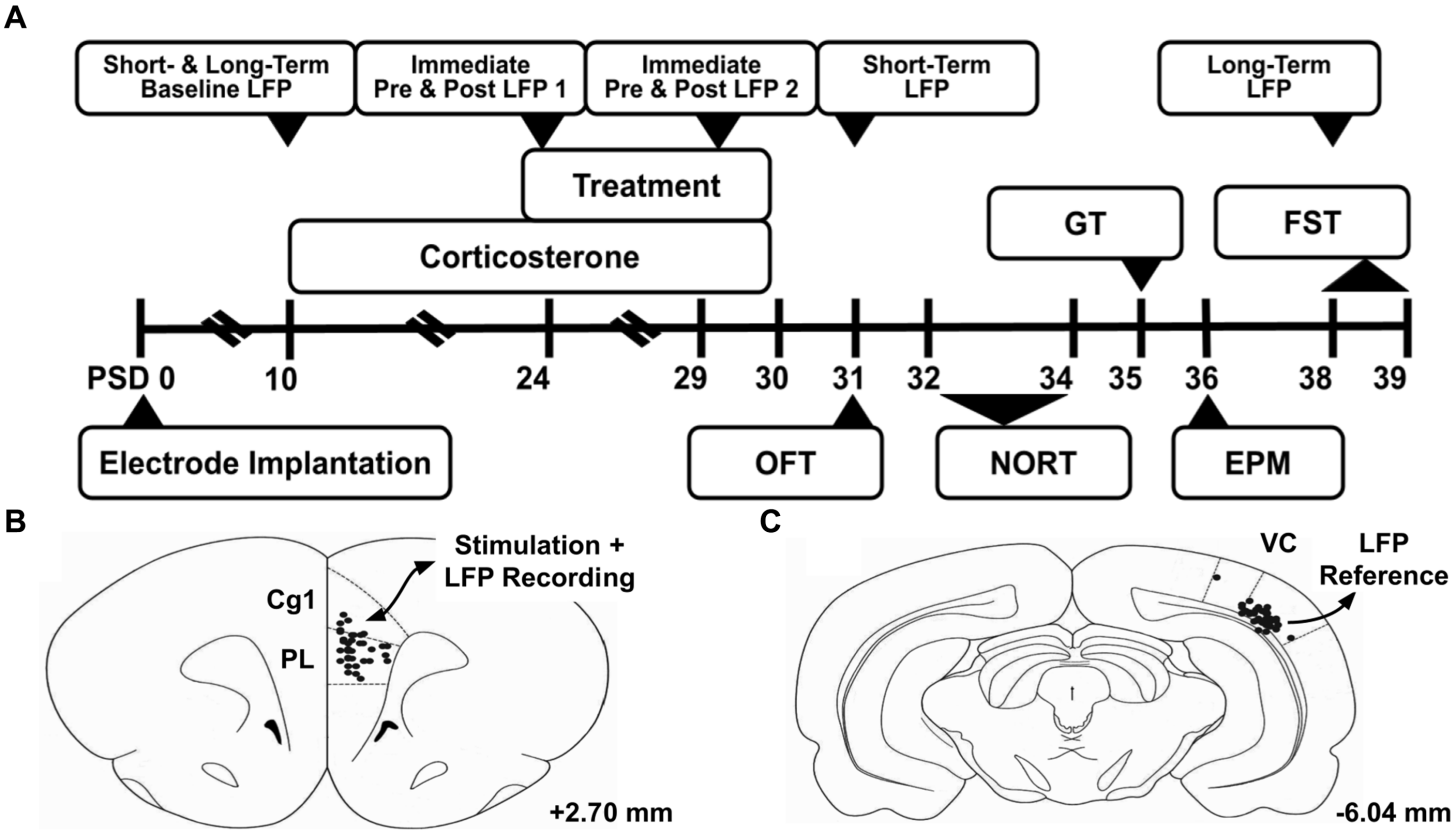
Experiment overview. **(A)** Timeline of experiment. **(B)** Electrode tip locations are marked with black dots in the region of interest, the medial prefrontal cortex. This region was the target of the deep brain stimulation treatment, and studied through local field potential (LFP) recordings throughout the experiment. **(C)** Electrode tip locations of the reference electrode in the lateral visual cortex (VC). Atlas illustrations adapted from Paxinos and Watson (2007), and the given coordinates are referenced from bregma. PSD, Post Surgery Day; LFP, Local Field Potential; OFT, Open Field Test; NORT, Novel Object Recognition Test; GT, Groom Test; EPM, Elevated Plus Maze; FST, Forced Swim Test; Cg1, Cingulate Cortex Area 1; PL, Prelimbic Cortex; VC, Visual Cortex.

An overview of the experimental design is given in Figure 1A. After acclimation, the animals underwent electrode implantation surgery. Under general anesthesia (2–4% isoflurane in O_2_ carrier), a midline incision was made in the scalp. Using a digital stereotaxic frame, twisted bipolar tungsten electrodes (P1 Technologies, Roanoke County, Virginia) were inserted into the mPFC (ML: 0.6 mm relative to bregma, AP: 2.5 mm, DV: 4.0 mm) (Figure 1B) and lateral visual cortex (ML: 5.5 mm, AP: −6.0 mm, DV: 2.5 mm; Figure 1C; Paxinos and Watson, 2007). Six partial thickness holes were drilled into the skull, and six stainless steel autoclave-sterilized anchoring screws (#0–80) were threaded into the skull. The electrodes were then anchored in place using methyl methacrylate.

### 2.3 Pharmacology and stimulation

#### 2.3.1 Corticosterone depression model

On post-surgery days (PSD) 10–30, 40 mg/kg CORT (C2505, Sigma-Aldrich, Milwaukee, WI) was subcutaneously administered, except in the control group which received 5% DMSO/saline vehicle. Previous studies have demonstrated that this dose and duration induces depressive-like symptoms, such as increases in anhedonia, anxiety, and despair, decreases in evoked grooming behavior, impairments in declarative memory, and weight deficits (Sterner and Kalynchuk, 2010). Animals received the CORT dose after LFP recordings, treatment administrations, and behavioral tasks were completed for the day.

#### 2.3.2 Ketamine treatment

On PSD 24–30, 15 mg/kg ketamine hydrochloride (K2753, Sigma-Aldrich, Milwaukee, WI) were intraperitoneally administered to the CORT+KET and CORT+DBS+KET groups. This dose and duration was chosen based on previous studies (Garcia et al., 2008), which show a rescue of several depressive-like symptoms listed in the previous section. Saline vehicle was administered to the control, CORT, and CORT+DBS groups.

#### 2.3.3 Deep brain stimulation treatment

On PSD 24–30 the mPFC electrode of animals in the CORT+DBS and CORT+DBS+KET groups were connected to an isolated pulse stimulator (STG 4008, Warner Instruments LLC, Hamden, CT) via a twisted wire cable. Continuous electrical stimulation was delivered using the following stimulation parameters: 100 microsecond square wave pulses at 130 Hz and current of 80 μA for 30 min. These parameters were selected based on previous literature (Hamani and Nóbrega, 2010; Hamani et al., 2012), which demonstrated a rescue of several depressive-like symptoms listed in the CORT section. Animals in the CORT+DBS+KET group were administered the ketamine dose immediately before stimulation began.

### 2.4 Behavioral tests and weight

#### 2.4.1 Open Field Test (OFT)

Non-specific changes in locomotor activity after corticosterone, ketamine, and stimulation (Karatsoreos et al., 2010; Parise et al., 2013; Papp et al., 2022) can confound behavioral and electrophysiological results, therefore we measured average velocity in the Open Field Test on PSD 31. Animals were placed in an open, square arena (50 × 50 cm) and allowed to explore for 10 min. The average speed of locomotion was calculated using the video tracking software TopScan Lite (Clever Sys Inc., Reston, VA). Similarly, non-specific changes in grooming behavior can confound the Groom Test (Planchez et al., 2019), therefore the number of seconds spent spontaneously grooming during the OFT were evaluated.

#### 2.4.2 Novel Object Recognition Test (NORT)

Cognitive dysfunction, including deficits in memory, often occur in depression (American Psychiatric Association, 2013). To measure these, we employed the Novel Object Recognition Test (NORT). In this test, a higher proportion of time spent with a novel object, compared to a familiar object, is interpreted as representing functional object recognition memory (Antunes and Biala, 2012). The methodology employed is in the Supplementary materials. In short, we calculated the average percent change in the Discrimination Index (DI) (difference in time exploring the novel versus familiar object, divided by the total time exploring both objects) between a familiarization day and two test days.

#### 2.4.3 Groom Test (GT)

As healthy rodents groom themselves thoroughly in response to soiling of their coats, reduced evoked grooming in the Groom Test (GT) has been interpreted to represent apathy, a core symptom of depression (Kennedy, 2008; Planchez et al., 2019). The animals performed the GT on PSD 35, their dorsoposterior coats were sprayed with a 10% sucrose solution, and were observed for 10 min in the OFT/NORT arena. Video recordings were evaluated for the number of seconds spent grooming.

#### 2.4.4 Elevated Plus Maze (EPM)

Anxiety is highly comorbid with depression (American Psychiatric Association, 2013), and has been shown to be decreased after ketamine treatment, though whether it relates to its therapeutic efficacy for depression is unclear (Hartland et al., 2023). Therefore, on PSD 36, the Elevated Plus Maze (EPM) was used to measure anxiety, where a higher proportion of time in the innately fear-inducing “open” arms is interpreted as less baseline anxiety (Planchez et al., 2019). The maze and methodology are described in the Supplementary materials.

#### 2.4.5 Forced Swim Test (FST)

Despair, the second core symptom of depression beside apathy (Kennedy, 2008), is typically measured in rodents via the Forced Swim Test (FST), where increased immobile time in the test trial putatively represents despair (Planchez et al., 2019). On PSD 38, the animals were habituated to the FST testing chamber (glass cylindrical tub 50 cm tall, 25 cm diameter, filled with 21°C water 40 cm high) for 10 min. On PSD 39, the animals were tested for 5 min. A video recording of these five minutes was analyzed to determine the number of seconds spent immobile. After each trial the animals were placed in a heated chamber, dried, and monitored for full recovery.

#### 2.4.6 Weight

Weight deficits are another common symptom of depression (American Psychiatric Association, 2013). Furthermore, similar to locomotor activity, non-specific changes in weight can confound behavioral results. Therefore, weights were measured at the “pre” CORT/treatment time point PSD 8–10, as well as the “post” time points PSD 30–32 and 37–39. Relative weights were calculated by dividing an animal’s weight at the “post” time points by the “pre” time point. The average of three days was used due to high variability in weight caused by the SPT fasts.

### 2.5 Local field potential recordings and preprocessing

To provide a baseline from which to track the short- and long-term oscillatory changes induced by chronic CORT and treatment, a 10 min local field potential (LFP) recording of the mPFC was taken on PSD 10, prior to the first CORT dose, using a Cheetah Digital Lynx SX Data Acquisition System (Neuralynx, Bozeman, MT). To investigate long-term changes following the last CORT and/or treatment administration on PSD 30, 10 min LFP recordings on PSD 31 and PSD 38 were taken. To investigate the immediate oscillatory effects induced by treatment administration, 10 min baseline and 20 min post-treatment recordings were taken on PSD 24 and PSD 29, after stimulation was turned off.

These recordings were referenced against an arbitrary cortical region, the lateral secondary visual cortex (V2L). To standardize recording quality, segments at least 3 s long and containing less than 2% noise (values above a threshold determined via visual inspection) were analyzed. Power spectral density plots were generated using the pwelch() function in MATLAB R2019b (MathWorks, Natick, MA). All recordings occurred in a designated arena (30 cm x 46 cm x 19 cm) while the animals were awake, during the light phase of the animals’ circadian rhythms.

### 2.6 Spectral parameter and sample entropy calculations

To parameterize the power spectral density plots generated for each recording, the MATLAB wrapped spectral parameterization (FOOOF) algorithm (version 1.1.0) (Donoghue et al., 2020) was applied to the raw signal segments with the following settings: peak width limits: [0.5, 25], max number of peaks: 5, and aperiodic mode: ‘no knee’, across the frequency range 1–50 Hz. This produced power spectral density plots for the periodic and aperiodic components of the original plot. Subtracting the aperiodic component from the periodic generated an aperiodic-adjusted power spectral density plot. We found consistent peaks in the theta range (5–10 Hz) and extracted the average center frequency and width of the peaks detected in each range, weighted by the height. Peaks in the low gamma range (20–50 Hz) were detected less consistently and with more variability in peak parameters. We extracted the average center frequency and width of the peaks, weighted by the width. Theta and low gamma power were calculated by summing the area under the aperiodic-adjusted power spectral density plot curve within each range. We also extracted the aperiodic parameters, the offset (also known as total broadband power) and the exponent (the slope of the extracted aperiodic power spectral density plot), across the 1–50 Hz range.

Additionally, sample entropy, a measure of signal irregularity, was calculated by applying the sampen() MATLAB function to the 1–50 Hz bandpass-filtered signal, divided into samples of length *n* = 5,000 (Martínez-Cagiga, 2018). Sample entropy measures the negative natural logarithm of the conditional probability that a sequence that matches for *m* points will continue to match at the next point, within a tolerance *r*. Therefore, a high sample entropy value signifies higher irregularity or unpredictability in the data because it reflects a low probability of finding matching sequences. Because the value of sample entropy depends heavily on the choice of *m* and *r* parameters, we calculated its value across the ranges of *m* (2, 3, 4) and *r* (0.1, 0.15, 0.2, 0.25) recommended when studying biological systems (Molina-Picó et al., 2011). As one aim of this study is to find electrophysiological biomarkers that can differentiate between healthy, depression-like, and remission-like neural activity, we then compared each combination of parameters (Supplementary Figures S1, S4) to select the one with the most between- and within-group differences.

Percent change in spectral parameters and sample entropy was calculated by subtracting the value obtained during baseline recording (pre-CORT: PSD 10; pre-treatment: PSD 24 and 29) from the value in the recording of interest (post-CORT: PSD 31 and 38; post-treatment: PSD 24 and 29), dividing by the value of the baseline recording, and multiplying by 100.

### 2.7 Statistical analysis

Statistical analyses were performed using R 4.2.2 (R Core Team, 2022). Two-sample t-tests and/or ANOVA were used to compare between groups, while one-sample tests revealed if a group changed significantly relative to their baseline (percent change/mu = 0). Standard parametric tests (one- or two sample t-test, ANOVA, Pearson’s r) were used when assumptions of normality and homoscedasticity were met and unless otherwise stated, while non-parametric tests (one- or two-sample Wilcoxon rank sum test, aligned rank transformed (ART) ANOVA, Spearman’s ρ/rho) were used otherwise. Multiple test corrections were done with the False Discovery Rate method. For all tests, alpha was set to 0.05 (two-tailed). Experimenters were blinded to the animal’s condition during behavioral test evaluation.

### 2.8 Histology

On PSD 39, rats were euthanized by anesthesia (Isoflurane) and were transcardially perfused with 100 mL of 0.1 M sodium phosphate buffer saline (PBS, pH −7.4), followed by 50 mL of 4% paraformaldehyde (pH 7.4). Brains were extracted and stored in 4% paraformaldehyde at 4°C. Serial coronal sections were cut at 100-μm thickness with a vibratome (Leica VT 1200; Leica Biosystems, Buffalo Grove, IL) starting at +3.8 mm Bregma and ending at −6.50 mm Bregma. Sections in the vicinity of electrodes were mounted onto 0.1% gelatin-subbed slides and stained with NeuroTrace 530/615 Red Fluorescent Nissl Stain (N21482, ThermoFisher Scientific, Waltham, MA, USA) to confirm proper placement (Figures 1B,C).

## 3 Results

### 3.1 Chronic CORT and treatment induced differences in depression-related behavioral measures and weight

One-way ANOVAs were done on the results of the behavioral assay to determine whether there were differences between groups. Significant group effects were found in the Groom Test [*F*(4,1) = 2.75, *p* = 0.049, ART ANOVA], Forced Swim Test [*F*(4,1) = 3.47, *p* = 0.017, ART ANOVA], relative weight on PSD 31 [*F*(4,1) = 12.85, *p* < 0.001] and PSD 38 [*F*(4,1) = 6.31, *p* < 0.0001], and locomotion in the OFT [*F*(4,1) = 2.99, *p* = 0.031]. No significant group effects were observed in spontaneous grooming in the OFT, the Elevated Plus Maze, or the Novel Object Recognition Test.

#### 3.1.1 Chronic CORT administration induced apathy-like symptoms in the GT, which was fully rescued by separate repeated DBS or ketamine treatment, but not by the combinatorial treatment

**Figure 2:**
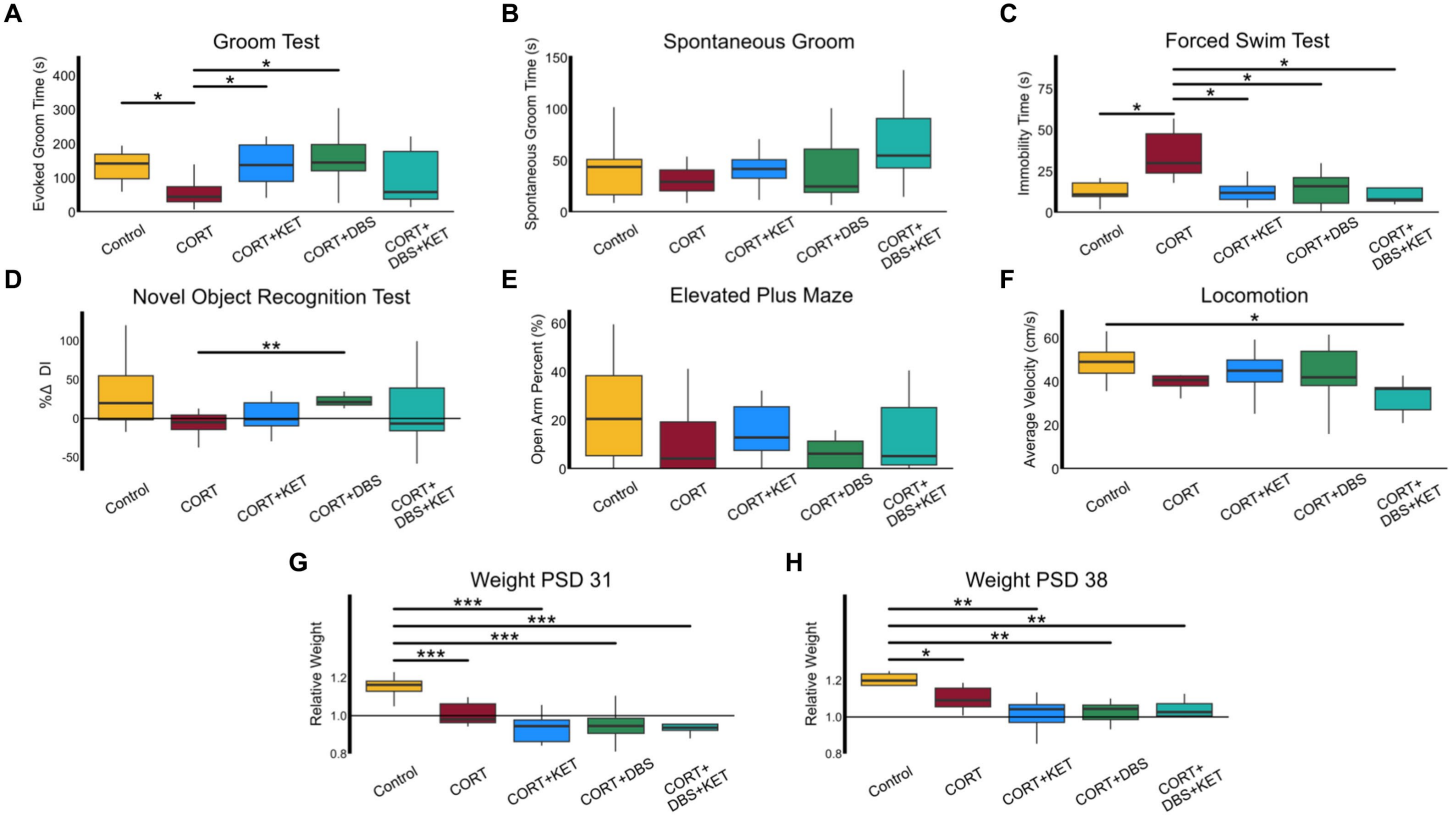
Behavioral and weight changes. **(A)** The CORT group (*n* = 8) spent significantly less time grooming than the control group (*n* = 8) indicating apathy-like symptoms were induced in the CORT group. The groups that received DBS (*n* = 8) or ketamine treatment (*n* = 9) separately spent significantly more time grooming than the CORT group, representing a rescue of apathy-like symptoms. The CORT+DBS+KET group (*n* = 9) did not significantly differ from the CORT group, suggesting apathy-like symptoms were not rescued. **(B)** Non-specific alterations in grooming activity can confound the GT, however, spontaneous grooming behavior in the OFT did not differ between groups. **(C)** The CORT group spent more time immobile in the FST compared to the control and treatment groups, indicating despair-like symptoms had been induced in the CORT group, and rescued by all treatment strategies. **(D)** In the NORT, the percent change in Discrimination Index (DI), a measure of object recognition memory, was greater in the CORT+DBS group than the CORT group. **(E)** There were no differences in open arm time percentage in the EPM, a measure of anxiety-like behavior. **(H)** The average velocity of the CORT+DBS+KET group in the OFT was lower than the control group. Relative weight in all groups administered CORT was less than in the control group on **(F)** PSD 31 and **(G)** PSD 38, indicating depression-like weight disturbances were induced, and were not rescued by any treatment strategy. *p < 0.05 in FDR-corrected two-sample tests; **p < 0.01; ***p < 0.001; PSD, Post Surgery Day; CORT, Corticosterone; DBS, Deep Brain Stimulation; KET, Ketamine; GT, Groom Test; FST, Forced Swim Test; NORT, Novel Object Recognition Test; DI, Discrimination Index; EPM, Elevated Plus Maze; OFT, Open Field Test.

Specific behavioral differences between groups were explored through post-hoc tests, as shown in Figure 2. During the GT (Figure 2A), the CORT group spent significantly less time grooming than the control group (Supplementary Table S1, Row 1), indicating apathy-like symptoms had been induced. The groups that received DBS or ketamine treatment separately spent significantly more time grooming than the CORT group, representing a rescue of apathy-like symptoms. In contrast, the CORT+DBS+KET group did not significantly differ from the CORT group, suggesting apathy-like symptoms were not rescued. In addition, non-specific alterations in grooming activity can confound the GT, however, spontaneous grooming in the OFT did not differ between groups (Figure 2B).

#### 3.1.2 Chronic CORT administration induced despair-like behavior in the FST, which was rescued by chronic DBS, ketamine, and the combinatorial treatment

The CORT group spent more time immobile in the FST (Figure 2C), compared to the control group, indicating despair-like symptoms had been induced (Supplementary Table S1, Row 2). This behavior was rescued in all three treatment groups, as they spent less time immobile than the CORT group.

#### 3.1.3 Anxiety (EPM) and object recognition memory (NORT) measures were unaltered by chronic CORT, however DBS improved NORT performance

In the NORT (Figure 2D), the percent change in DI was greater in the CORT+DBS group than the CORT group (Supplementary Table S1, Row 3). This indicates an improvement in object memory, however, as there was no deficit in the CORT group compared to the control group, this does not represent a “rescue” of memory function. There were no differences in open arm time percentage in the EPM between the CORT and control or treatment groups, indicating that, as has been previously reported after chronic corticosterone (Bertholomey et al., 2022), comorbid anxiety-like symptoms were not induced (Figure 2E).

#### 3.1.4 Chronic CORT induced weight disruptions that were not rescued by any treatment, and long-term locomotor activity was only altered in the CORT+DBS+KET group

No groups differed from the CORT group in average velocity in the OFT (Figure 2F), though the CORT+DBS+KET group had lower average velocities than the control group (Supplementary Table S1, Row 4). This indicated that while chronic CORT, alone or in combination with repeated ketamine or mPFC DBS, did not induce a deficit in locomotion, a combination of all three reduced average velocity.

The average relative weight in the CORT group was less than in the control on PSD 31 (Figure 2G) and PSD 38 (Figure 2H), indicating depression-like weight deficits had been induced. The three treatment groups did not differ from the CORT group, and had lower relative weights than the control group on PSD 31 (Supplementary Table S1, Row 5) and PSD 38 (Supplementary Table S1, Row 6). Therefore none of the treatment strategies tested rescued these deficits in weight.

### 3.2 mPFC LFP spectral parameters and sample entropy were modulated immediately after treatment

**Figure 3:**
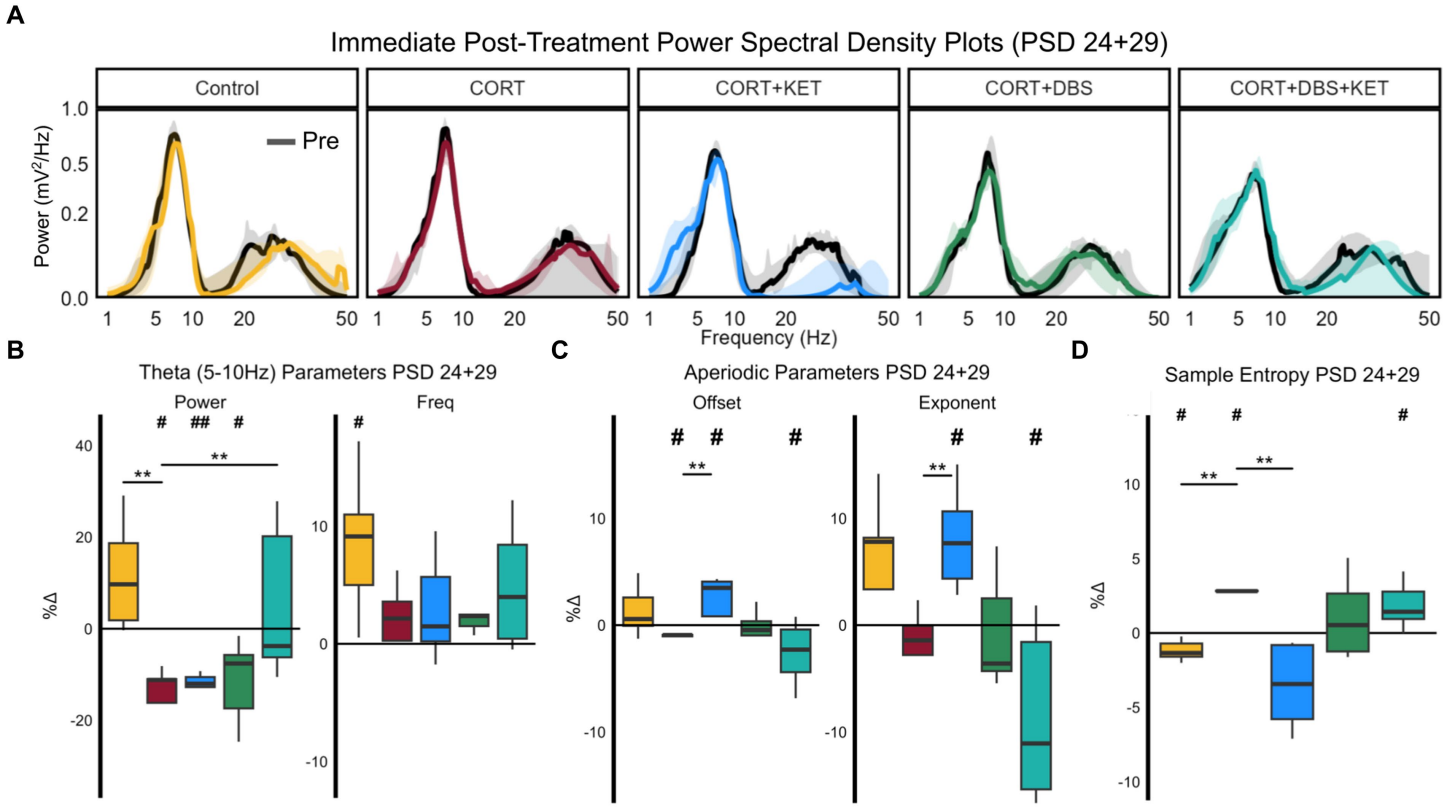
Changes in spectral parameters and sample entropy immediately post-treatment, PSD 24+29. **(A)** Median aperiodic-adjusted periodic power spectrum density plots of the local field potential recordings on PSD 24+29 immediately pre- (black) and post-treatment (colored) for each group. **(B)** Boxplots of the percent change in periodic theta (5–10 Hz) spectral parameters aperiodic-adjusted power (mV^2^/Hz) and peak frequency (Hz). Theta power significantly decreased relative to the pre-treatment baseline in the CORT, CORT+KET, and CORT+DBS groups (indicated by ‘#’). In the CORT group, this significantly differed from the control and CORT+DBS+KET groups (indicated by ‘*’). Theta peak frequency increased significantly in the control group. **(C)** Boxplots of the percent change in the aperiodic spectral parameters offset and exponent. The offset was significantly increased in the CORT+KET group relative to baseline and the CORT group, which decreased significantly from baseline. Similarly, the offset significantly decreased in the CORT+DBS+KET group relative to baseline. The exponent also increased significantly compared to baseline and the CORT group, and the CORT+DBS+KET group decreased relative to baseline. **(D)** Boxplots of the percent changes in sample entropy. Sample entropy significantly increased in the CORT group relative to the control and CORT+KET groups, and the control group decreased significantly relative to baseline. Sample entropy in the CORT+DBS+KET group also increased significantly relative to pretreatment baseline (*, #*p* < 0.05 in one/two sample tests; **, ##*p* < 0.01); CORT, Corticosterone; PSD, Post Surgery Day; DBS, Deep Brain Stimulation; KET, Ketamine; Freq, Peak Frequency.

To investigate the acute effects of treatment administration on mPFC LFP, we calculated the percent changes in spectral parameters and sample entropy immediately before and after treatment on PSD 24 and 29. The pre- (black) and post-treatment (colored) aperiodic-adjusted power spectrum density plots for each group is shown in Figure 3A. For spectral parameters, uncorrected two-way ANOVAs with group and day effects found significant group effects for the following spectral parameters: offset [*F*(4,1) = 4.60, *p* < 0.01, ART ANOVA], and exponent [*F*(4,1) = 4.21, *p* = 0.011, ART ANOVA]. Theta peak frequency [*F*(4,1) = 2.55, *p* = 0.056] and power [*F*(4,1) = 2.09, *p* = 0.095] showed trends strong enough to warrant further post-hoc analysis. No day effects were found, and there were no significant group x day interactions for any parameter, therefore, the average of both days was used for the post-hoc analyses. For sample entropy, two-way, multiple-test corrected ANOVAs found significant group effects for all parameter combinations. We selected the parameter combination *r* = 0.25 and *m* = 4 (Supplementary Figure S1) for further analysis [*F*(4,1) = 6.03, *p* < 0.01], as post-hoc tests revealed the strongest between- and within-group differences.

#### 3.2.1 Periodic parameters were modulated by CORT and all treatments

Theta power decreased relative to pre-treatment baseline on PSD 24+29 significantly in the CORT, CORT+KET, and CORT+DBS groups (Figure 3B; Supplementary Table S2, Row 1). For the CORT group, this differed significantly from the control and CORT+DBS+KET groups, which did not change. Meanwhile, theta peak frequency significantly increased relative to baseline in the control group, which differed trendwise from the CORT group which did not change.

In the control group, low gamma peak frequency increased significantly relative to baseline and trendwise relative to the CORT group (Supplementary Figure S2A). Meanwhile, low gamma peak width increased trendwise relative to the CORT group in the CORT+KET and CORT+DBS groups.

#### 3.2.2 Aperiodic parameters were modulated in both ketamine treated groups

The offset significantly increased in the CORT+KET group immediately after treatment on PSD 24+29 relative to both baseline and the CORT group, which decreased significantly relative to the pretreatment baseline (Figure 3C; Supplementary Table S2, Row 2). In contrast, the offset decreased trendwise in the CORT+DBS+KET group relative to baseline.

Changes in the exponent were very similar. The exponent increased in the control (trendwise) and CORT+KET group (significant) relative to the pretreatment baseline and the CORT group. In contrast, the exponent of the CORT+DBS+KET group decreased significantly compared to baseline and trendwise compared the CORT group.

#### 3.2.3 Sample entropy decreased in the control and CORT+KET group, while it increased in the CORT and CORT+DBS+KET group

Sample entropy significantly decreased in the control and CORT+KET groups relative to their baseline and the CORT group immediately after treatment on PSD 24+29 (Figure 3D; Supplementary Table S2, Row 3). Meanwhile, the CORT and CORT+DBS+KET group both significantly increased in sample entropy relative to their pretreatment baseline. The CORT and CORT+DBS+KET groups increased relative to baseline across all parameter combinations tested (Supplementary Figure S1), while the control group increased in 7 of 12 pairings. Furthermore, the control and CORT groups significantly differed in every pairing, while the CORT and CORT+KET groups differed in all but one.

### 3.3 mPFC spectral parameters and sample entropy were altered in the short- and long-term post-CORT and treatment

To explore the short- and long-term effects of CORT and treatment on mPFC LFP spectral parameters and sample entropy, we calculated the percent changes in these measures between a pre-CORT baseline on PSD 10, and two time points after the last CORT and treatment administration on PSD 30. By the short-term time point, PSD 31, one-way ANOVAs found group differences in theta peak frequency [*F*(4,1) = 2.90, *p* = 0.045] and the exponent [*F*(4,1) = 5.14, *p* < 0.01]. The PSD 10 (black) and PSD 31 (colored) aperiodic-adjusted power spectrum density plots for each group are shown in Supplementary Figure S3A. Post-hoc tests found that while theta peak frequency decreased in both the CORT and CORT+KET, the CORT+KET group decreased significantly more relative to the CORT group (Supplementary Figure S3B; Supplementary Table S2, Row 4). For the exponent, the control group increased trendwise relative to the CORT group (Supplementary Figure S3C; Supplementary Table S2, Row 5). Within-group tests found that relative to the pre-CORT baseline, the offset and exponent significantly decreased in the CORT+DBS group. A one-way ANOVA found no effect by group in sample entropy for any parameter combination by PSD 31.

**Figure 4:**
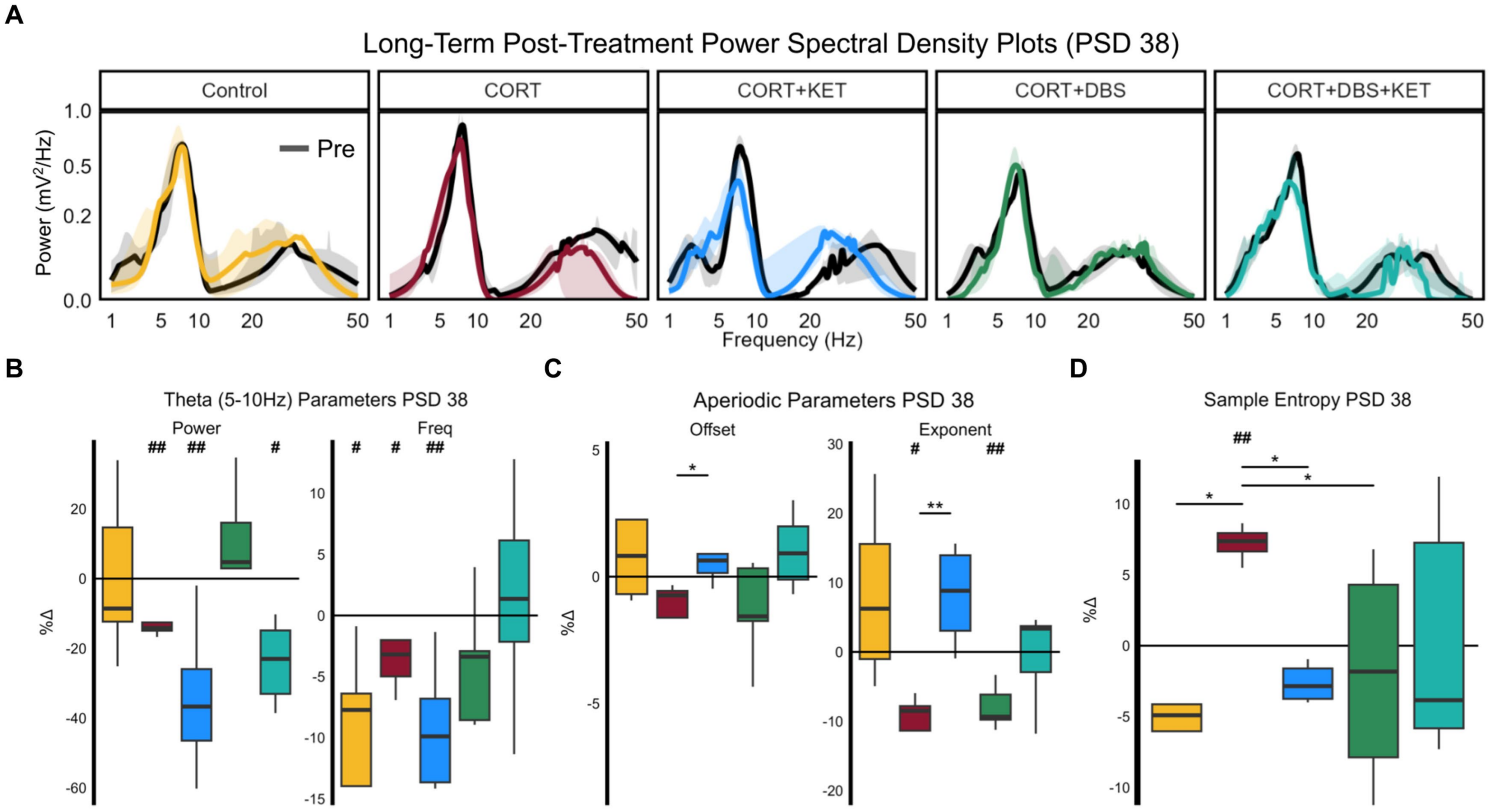
Changes in spectral parameters and sample entropy eight days post-treatment, PSD 38. **(A)** Median aperiodic-adjusted periodic power spectrum density plots of the baseline (black) local field potential recordings on PSD 10, and the long-term post-treatment recording on PSD 38 (colored). **(B)** Boxplots of the percent change in periodic theta (5–10 Hz) spectral parameters aperiodic-adjusted power (mV^2^/Hz) and peak frequency (Hz). Theta power decreased in the CORT, CORT+KET, and CORT+DBS+KET groups relative to baseline (indicated by ‘#’). In the control, CORT, and CORT+KET group, theta peak frequency decreased. **(C)** The offset and exponent increased significantly in the CORT+KET group relative to the CORT group. The exponent decreased relative to baseline in the CORT and CORT+DBS groups. **(D)** Boxplots of the percent change in sample entropy, which was significantly higher in the CORT group compared to the control, CORT+KET, and CORT+DBS groups, as well as the pre-CORT baseline (*, #*p* < 0.05 in uncorrected one/two sample tests; **, ##*p* < 0.01); CORT, Corticosterone; PSD, Post Surgery Day; DBS, Deep Brain Stimulation; KET, Ketamine.

By the long-term time point, PSD 38, uncorrected one-way ANOVAs found significant group effects in theta power [*F*(4,1) = 3.39, *p* = 0.026] and peak frequency [*F*(4,1) = 3.39, *p* = 0.026], as well as exponents [*F*(4,1) = 5.85, *p* < 0.01]. The PSD 10 (black) and PSD 38 (colored) aperiodic-adjusted power spectrum density plots for each group are shown in Figure 4A. For sample entropy, one-way ANOVAs found a trend in group effects for one parameter combination, *r* = 0.25 and *m* = 4 [Supplementary Figure S4; *F*(4,1) = 2.46, *p* = 0.075].

#### 3.3.1 Theta power, peak frequency, and peak width were modulated in the long-term by CORT, ketamine, and DBS

Theta power by PSD 38 decreased significantly in the CORT, CORT+KET, and CORT+DBS+KET groups relative to baseline (Figure 4B; Supplementary Table S2, Row 6). Theta power decreased trendwise more in the CORT+KET group compared to the CORT group. Furthermore, in control, CORT, and the CORT+KET group, theta peak frequency decreased significantly relative to baseline. Like power, theta peak frequency decreased trendwise more in the CORT+KET group compared to the CORT group.

#### 3.3.2 Low gamma parameters decreased in the CORT and CORT+KET groups

Low gamma power (trendwise), peak frequency (significant), and peak width (significant) decreased relative to baseline by PSD 38 in the CORT group (Supplementary Figure S2C; Supplementary Table S3, Row 6). Similarly, low gamma peak frequency decreased trendwise by PSD 38 in the CORT+KET group.

#### 3.3.3 The aperiodic parameters were modulated in the long-term by CORT and ketamine

The exponent significantly decreased by PSD 38 in the CORT and CORT+DBS groups (Figure 4C; Supplementary Table S2, Row 7) relative to pre-CORT baseline, while it increased trendwise in the CORT+KET group. For the CORT group, this differed significantly from the CORT+KET group, and trendwise from the control and CORT+DBS+KET groups. Furthermore, the offset decreased trendwise in the CORT group, which differed significantly from the CORT+KET group and trendwise from the CORT+DBS+KET group.

#### 3.3.4 Sample entropy was normalized by DBS or ketamine separately, but not combined

By PSD 38, sample entropy was significantly lower in the control, CORT+KET, and CORT+DBS groups compared to the CORT group (Figure 4D; Supplementary Table S2, Row 8), which had increased relative to its baseline. The CORT+KET group decreased trendwise from its baseline. It should be noted that while the control and CORT+KET groups differed from the CORT group in all 12 parameter combinations, the CORT and CORT+DBS groups only differed in one parameter combination (Supplementary Figure S4). The CORT group increased significantly from baseline in seven parameter combinations.

### 3.4 Electrophysiological measures and cognitive behavioral measures correlated with the Groom and Forced Swim Tests

We then explored the linear relationships between electrophysiological and behavioral measures throughout the experiment. The *p*-values of Pearson or Spearman’s linear correlation coefficients were calculated for several types relationships. To best investigate which measures related to the depression- and remission-like differences between the CORT group and the control or treatment groups, we pooled the results of each group with those of the CORT group to create a “group pool.” Only measures that significantly differed from baseline or from the CORT group were tested for correlation.

#### 3.4.1 Immediate, short- and long-term post-treatment electrophysiological and cognitive changes correlated with depression- and remission-like behavior

**Figure 5:**
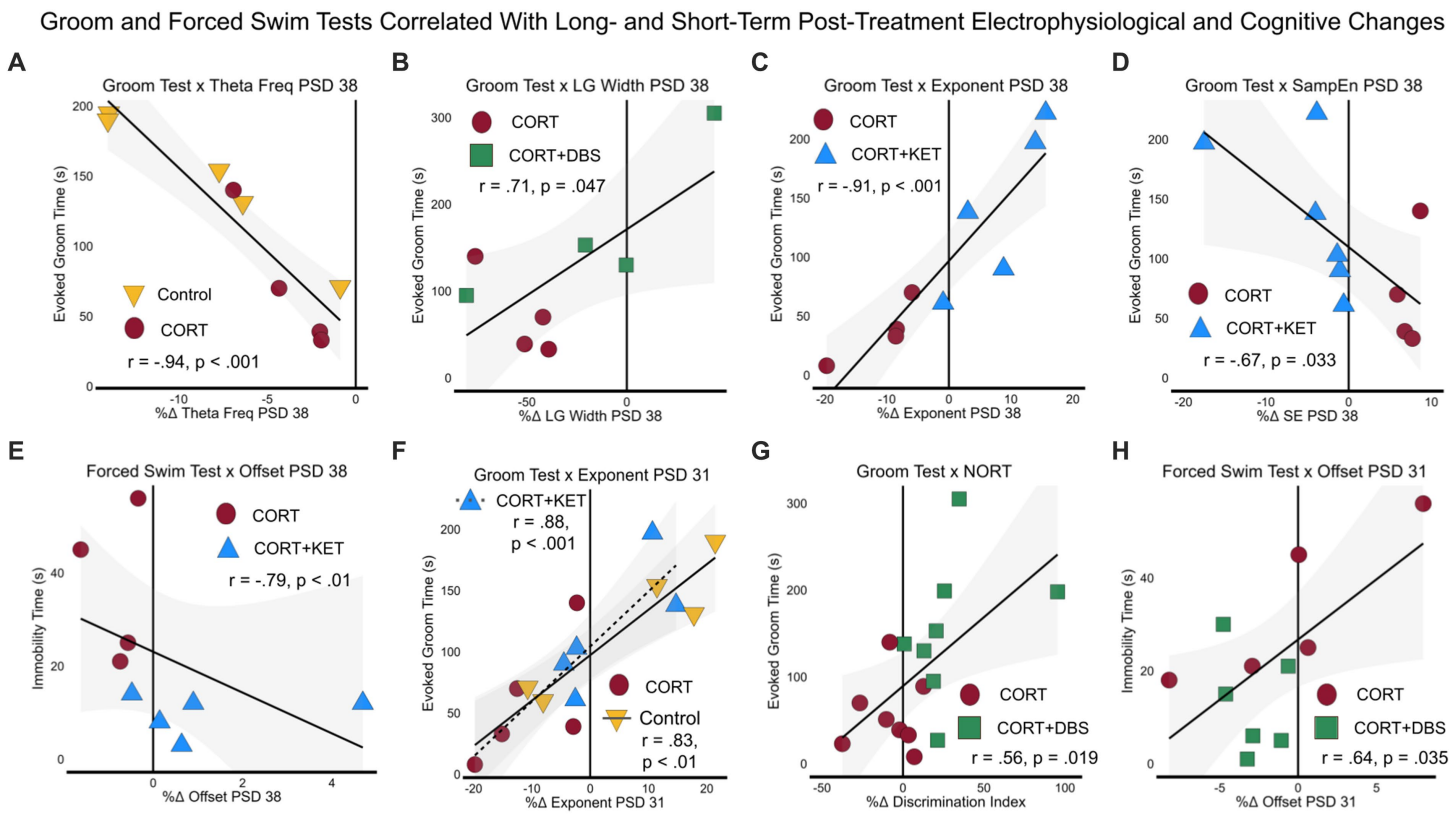
Linear regressions and correlation coefficients for the relationships between depression-related behavior and sustained post-treatment electrophysiological or cognitive changes. Depression-related behavioral measures GT (PSD 35) and FST (PSD 39) were correlated with short- (one day after last treatment, PSD 31) and long-term (eight days after last treatment, PSD 38) electrophysiological or cognitive (NORT, PSD 32–34) changes. Each group was pooled with the CORT group to create a “group pool.” **(A)** Theta peak frequency on PSD 38 negatively correlated with the GT in the control group pool. **(B)** In the CORT+DBS group pool, low gamma peak width on PSD 38 positively correlated with the GT. For the CORT+KET group pool on PSD 38, **(C)** the aperiodic exponent positively and **(D)** sample entropy negatively correlated with the GT, while **(E)** the aperiodic offset negatively positively correlated with the FST. **(F)** In both the control and CORT+KET group pools, the exponent on PSD 31 positively correlated with the GT. **(G)** In the CORT+DBS group pool, the NORT positively correlated with the GT, while **(H)** the offset on PSD 31 positively correlated with the FST. PSD, Post Surgery Day; CORT, Corticosterone; DBS, Deep Brain Stimulation; KET, Ketamine; GT, Groom Test; FST, Forced Swim Test; NORT, Novel Object Recognition Test; DI, Discrimination Index; Theta Freq, Theta Peak Frequency; SampEn/SE, Sample Entropy; LG, Low Gamma.

We first explored the relationship between the depression-related measures, the Groom Test (GT) on PSD 35 and the Forced Swim Test (FST) on PSD 39, and the long-term electrophysiological changes on PSD 38. Electrophysiological correlates of behavior at this time parallel clinical biomarkers for steady-state depression or remission, given the extended time since the last CORT or treatment administration (eight days). Furthermore, as this is the time point closest to the GT and FST, these electrophysiological correlates may provide evidence of the mechanism underlying the long-term depression-related behavioral effects of CORT and the treatment strategies. For the control group pool, healthy performance in the Groom Test correlated with the increase in theta peak frequency in the control group (Figure 5A; Supplementary Table S3, Row 1). In the CORT+DBS group pool, remission-like performance in the Groom Test correlated with the increase in low gamma peak width in the treated group compared to the CORT group (Figure 5B). In the CORT+KET group pool, the Groom Test correlated with the increase in the exponent (Figure 5C) and offset, as well as the decrease in sample entropy (Figure 5D), in the CORT+KET group relative to the CORT group and its baseline. In addition, remission-like performance in the Forced Swim Test correlated with the increase in offset compared to the CORT group (Figure 5E).

Similarly, the second set of correlations examined the relationships between the depression-related measures (FST, GT), and short-term (PSD 31) electrophysiological and cognitive changes. In the control group pool, correlates at this time point parallel clinical biomarkers that track the early stages of depression after exposure to prolonged stress. In this pool, the increased exponent in the control group compared to the CORT group correlated with the GT (Figure 5F; Supplementary Table S3, Row 2). In contrast, in the treatment group pools, correlates at this time point parallel clinical biomarkers measured one day after treatment offset that may predict long-term remission. For the CORT+KET group pool, the increase in the exponent in the treated group also correlated with the GT (Figure 5F), while in the CORT+DBS group pool the improvement in the object memory task, the NORT, in the treated group correlated with the GT (Figure 5G). In addition, the decreased offset of the CORT+DBS group correlated with the FST (Figure 5H).

**Figure 6:**
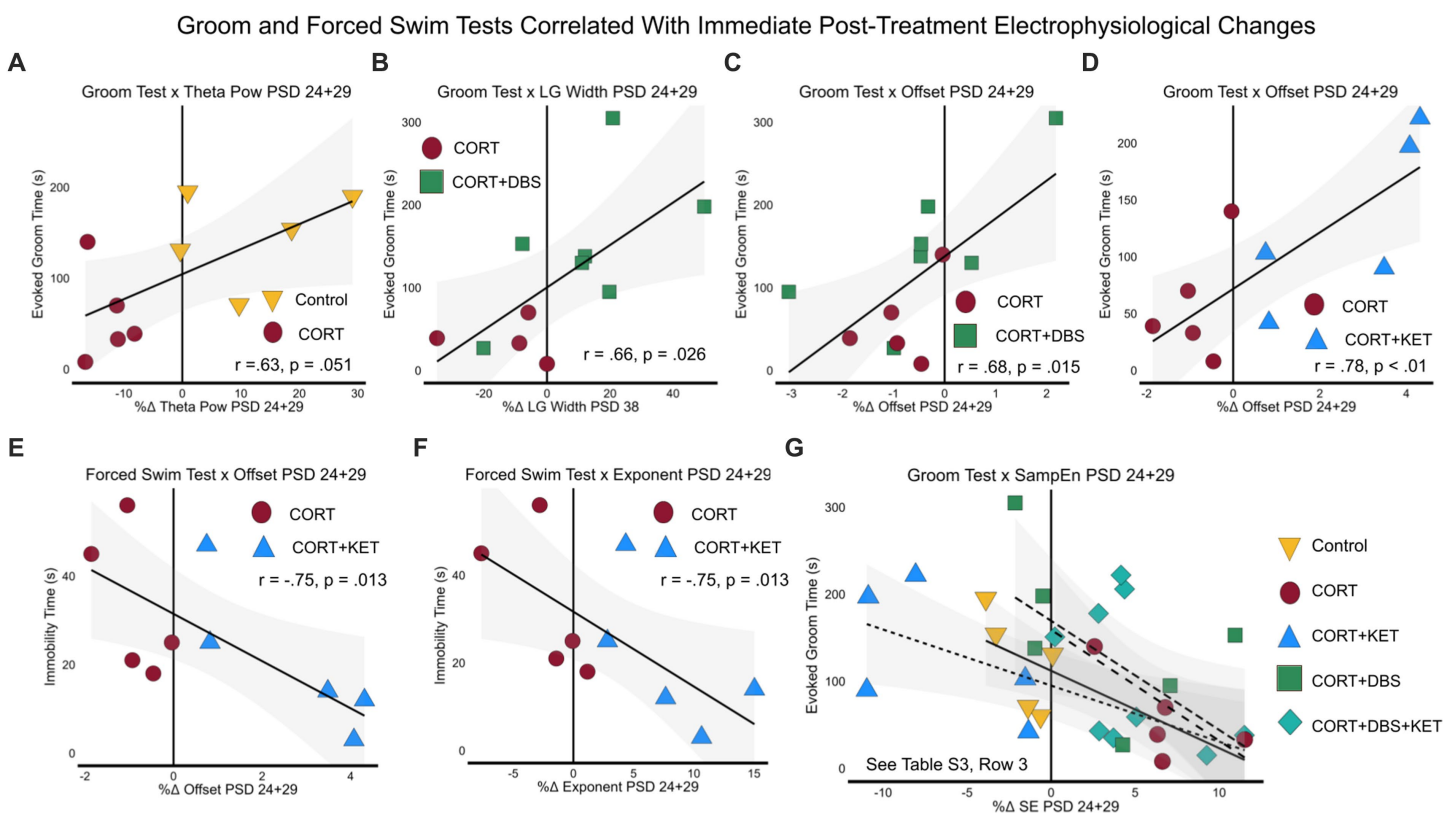
Linear regressions and correlation coefficients for the relationships between long-term depression-related behavior and immediate post-treatment electrophysiological measures. Depression-related behavioral measures GT (PSD 35) and FST (PSD 39) were correlated with significant immediate post-treatment (PSD 24+29) electrophysiological changes. Each group was pooled with the CORT group to create a “group pool.” **(A)** Aperiodic-adjusted theta power positively correlated with the GT in the control group pool. In the CORT+DBS group pool, **(B)** low gamma peak width and **(C)** the aperiodic offset correlated with the GT. In the CORT+KET group pool, **(D)** the offset also correlated with the GT, while the FST correlated negatively with both **(E)** the offset and **(F)** the aperiodic exponent. **(G)** Sample entropy negatively correlated with the GT in all four group pools. PSD, Post Surgery Day; CORT, Corticosterone; DBS, Deep Brain Stimulation; KET, Ketamine; GT, Groom Test; FST, Forced Swim Test; NORT, Novel Object Recognition Test; DI, Discrimination Index; Theta Pow, Theta Power; SampEn/SE, Sample Entropy; LG, Low Gamma.

We then related the immediate electrophysiological changes post-treatment (PSD 24+29) with the depression-related GT and FST behavioral tasks. Here, the control group pool correlates are intended to represent biomarkers of the chronically-stressed mPFC dysfunctionally responding to acute stress, in this case the mild stress of restraint, intraperitoneal saline injection, and sham stimulation. In this group pool, we see that the increase of theta power in the control, compared to the decrease of the CORT group, correlated with their GT performance (Figure 6A; Supplementary Table S3; Row 3). Meanwhile, in the treatment group pools, these correlates represent biomarkers measured immediately after treatment administration that may predict long-term remission. In the CORT+DBS group pool, the increase in low gamma peak width (Figure 6B) and offset (Figure 6C) in the treated group, compared to the CORT group, correlated with the GT. Similarly, in the CORT+KET group pool, the increase in the offset relative to the CORT group and the baseline in the treated group also correlated with the GT (Figure 6D). In addition, the FST correlated with both the exponent (Figure 6E) and the offset (Figure 6F). Finally, in all four group pools, sample entropy immediately after treatment correlated with the GT (Figure 6G).

#### 3.4.2 Putative electrophysiological and cognitive biomarkers correlated with one another across time

We then investigated whether the electrophysiological and behavioral biomarkers of depression-related behavior were correlated across the experiment, as this could hint at potential neural mechanisms of action for CORT and the treatment strategies. For each group pool, we first correlated the electrophysiological biomarkers from the long-term time point PSD 38, which have the potential to be causally related to performance on the GT and FST, with electrophysiological changes at the short-term post-treatment time point PSD 31. Those correlated PSD 31 measures were then correlated with the measures taken immediately after treatment on PSD 24+29. In the control group pool, the increase in theta peak frequency in the control group on PSD 38 correlated with the increase in the exponent in the control group on PSD 31 (Supplementary Figure S5A; Supplementary Table S3, Row 4). In turn, the increase in the exponent of PSD 31 correlated with the increase in theta power immediately after sham treatment on PSD 24+29 (Supplementary Figure S5B; Supplementary Table S3, Row 5). In the CORT+KET group pool, the decreased sample entropy in the treated group compared to the CORT group on PSD 38 correlated with the decrease in theta peak frequency on PSD 31 (Supplementary Figure S5C). In turn, the decrease in theta peak frequency on PSD 31 correlated with the decrease in sample entropy (Supplementary Figure S5D) and increase in offset and low gamma peak width immediately after treatment on PSD 24+29. For the same group pool, the decreased exponent (Supplementary Figure S5E) and offset, as well as the increased sample entropy (Supplementary Figure S5F) on PSD 38 in the CORT+KET group correlated with the increased exponent on PSD 31. In turn, the increased exponent on PSD 31 correlated with the decreased sample entropy immediately after treatment on PSD 24+29 (Supplementary Figure S5G). Finally, in the CORT+DBS group pool, improved performance in the NORT in the treated group correlated with the immediate increase in low gamma peak width immediately after treatment (Supplementary Figure S5).

#### 3.4.3 Depression-related weight deficits correlated with sample entropy and the exponent

To investigate the relationship between mPFC electrophysiological measures and the weight deficits observed in the CORT group, we performed similar analyses as in the first two sections for the control group pool. Decreased relative weight at the long-term time point on PSD 38 correlated with a decreased exponent on PSD 38 in the CORT group (Supplementary Figure S6A; Supplementary Table S3, Row 6). This decrease on PSD 38 correlated with a decrease in the exponent at the short-term time point (Supplementary Figure S6B). In turn, as described in section 4.4.2, the increase in the exponent on PSD 31 correlated with the increase in theta power immediately after sham treatment. Meanwhile, weight deficits on PSD 31 correlated with the decrease in the exponent (Supplementary Figure S6C) and increase in sample entropy (Supplementary Figure S6D) immediately after treatment.

#### 3.4.4 Non-specific motor or metabolic deficits did not confound the depression-related behavioral tasks

To ensure that differences in weight and locomotion did not confound our measures of depression-like behavior, we correlated each pool’s relative weights on PSD 38, and their average velocity in the OFT, with evoked groom and immobility time. While locomotion correlated with the GT in the control group pool (Supplementary Table S3, Row 7), there was no significant difference in locomotion between these two groups (Figure 2H). Therefore, this factor could not have driven the differences between groups in the GT. For the CORT+DBS+KET group pool, we also correlated locomotion with electrophysiological measures, however there were no correlations that explained the decreased locomotion in the treatment group. There were no significant correlations between weight and behavior. Overall, these findings are evidence that the deficits observed in the GT and FST were not due to non-specific motor or metabolic deficits.

#### 3.4.5 Sample entropy correlated with the offset, exponent, theta power, and low gamma peak width

As the neural underpinnings of sample entropy are not well understood, we tested whether sample entropy significantly correlated with spectral parameters. In general, sample entropy correlated consistently with the exponent and offset, and at times with theta power and low gamma peak width (Supplementary Figure S6, Row 2; Supplementary Table S3, Row 8/9).

## 4 Discussion

### 4.1 Separately, but not combined, DBS and ketamine rescued depression-like behavioral performance induced by CORT, which correlated with changes in mPFC LFP and cognitive measures across time

The aim of this experiment was to investigate the electrophysiological correlates of depression- and remission-like effects of corticosterone, ketamine, and mPFC DBS in rats. Given the key role of the mPFC dysfunction in driving depression symptoms (Pizzagalli and Roberts, 2022), these correlates are not only potential biomarkers for depression severity and treatment efficacy but they also may point at the mechanisms underlying depression- and remission-like behavior. Our stress-based depression model, chronic corticosterone administration, induced apathy-like behavioral performance in the Groom Test, despair-like behavior in the Forced Swim Test, and depression-like weight deficits. While remission-like performance on the FST was induced by ketamine and/or DBS, only ketamine or DBS separately rescued performance on the GT. To find biomarkers of these depression-related measures, we correlated them with changes in mPFC LFP at three time points post-treatment: immediate (after first and sixth treatment, PSD 24 & 29), short-term (one day after last treatment, PSD 31) and long-term (eight days after last treatment, PSD 38).

#### 4.1.1 Depression-like behavioral and weight effects of chronic CORT correlated with changes in the exponent, sample entropy, and theta parameters

Eight days after chronic CORT administration, we found that a long-term increase in theta peak frequency in the CORT group, compared to the control group, correlated with apathy-like behavior in the GT. At the same time point, a decrease in the exponent correlated with concurrent weight deficits, while earlier, at the short-term, one day post-CORT time point, a decreased exponent in the CORT group correlated with the GT. Together, these periodic and aperiodic markers represent potential biomarkers for early and steady-state depression symptoms after chronic stress. Further, when considering the neural underpinnings of these electrophysiological measures, our findings may point toward the potential long-term mechanisms of corticosterone in inducing depression-like behavioral and weight symptoms. The current understanding of steady-state depression etiology is the hyperactivity and excitability of the mPFC, which drives excessive long-range inhibitory signals to regions involved in motivation, cognition, reward, and emotion (Pizzagalli and Roberts, 2022). Therefore, since a decreased exponent represents decreased inhibition (Lombardi et al., 2017), and chronic stress decreases inhibitory GABAergic transmission in male rodents (Ghosal et al., 2020), these findings support the theory that corticosterone recapitulates the stress-based depression-inducing hyperexcitation of the mPFC (Sterner and Kalynchuk, 2010; Wilber et al., 2011; Arnsten et al., 2023). They also agree with *in-silico* findings regarding decreased exponent values in a inhibitory interneuron knock-down depression model (Guet-McCreight et al., 2024). While the potential role of aberrantly increased theta peak frequency in the depressive symptoms is unclear, we speculate that since hyperactivation of the hippocampal to mpFC pathway, which operates at the theta frequency (Padilla-Coreano et al., 2019), has been correlated with depression severity in humans (De Kwaasteniet et al., 2013; Sambataro et al., 2014) and rodent models (Airan et al., 2007), and the hippocampus operates at a higher theta frequency than the mPFC (Biskamp et al., 2017), perhaps the increase in peak frequency represents the pathway’s depression-related hyperconnectivity. However, more work will need to be done to understand the meaning of this potential biomarker.

While the short- and long-term time points represent steady-state depression-like conditions, electrophysiological markers at the immediate time point on PSD 24+29 may represent the depression-inducing, aberrant response of the mPFC to stress (restraint and sham treatment). At this time point, an immediate decrease in theta power and increase in sample entropy in the CORT group correlated with apathy-like performance in the GT, and each other. Sample entropy is a measure of signal irregularity and complexity, and has been shown to correlate with a region’s degree of functional activity, processing, and connectivity (Wang et al., 2018). It has also been demonstrated to increase in frontal EEG electrodes in depression patients (Čukić et al., 2020; Lin et al., 2020). Meanwhile, aperiodic-adjusted theta power has been shown to decrease in the mPFC when rats are in a perceived “safe” environment (Adhikari et al., 2010). Therefore, similar to the steady-state decrease in mPFC inhibition discussed previously, we hypothesize that the increase in functional activity and decrease in safety-related theta power during mild stress was indicative of corticosterone’s depression-inducing hyperactivation of the mPFC (Sterner and Kalynchuk, 2010; Wilber et al., 2011; Arnsten et al., 2023). Furthermore, we found that the exponent and theta peak frequency on PSD 38 correlated with the exponent on PSD 31, which in turn correlated with the exponent immediately after treatment on PSD 24+29. This consistent and interrelated thread of electrophysiological indicators within the mPFC hints at potential relationships between them, however, causality can not be inferred from the present study.

#### 4.1.2 The remission-like behavioral effects of ketamine correlated with changes in the exponent, offset, and sample entropy

After ketamine treatment alone, we found that a long-term decrease in sample entropy, and an increase in the exponent and offset, significantly correlated with remission of apathy-like behavior in the GT, and despair-like behavior in the FST. At the short-term post-treatment time point PSD 31, one day after the last ketamine treatment, the GT again correlated with an increase in the exponent relative to the CORT group. This was on par with the increase in the control group, which also correlated with the GT. Alone, these findings indicate these three measures may function as biomarkers for long-term treatment efficacy after ketamine. However, when coupled with our growing understanding of the neural dynamics they represent, these measures may also point toward potential mechanisms of ketamine’s therapeutic action. As previously discussed, the aperiodic exponent and offset have been correlated with inhibition, and sample entropy has been correlated with functional activity and connectivity. Meanwhile, excessive functional activity and connectivity of the mPFC is central to theories regarding the etiology of depression and CORT-based depression models. Therefore, we hypothesize that the increase in inhibition and decrease in function, in line with the control group, potentially represent ketamine exerting its long-term therapeutic effects by “normalizing” mPFC hyperactivity. Indeed, ketamine increases the inhibitory tone of the mPFC of rats 24 h post-treatment (Yin et al., 2021; Mingardi et al., 2023), and reduces the functional connectivity of the human prefrontal cortex for up to two weeks post-treatment (Chen et al., 2019; Siegel et al., 2021). Therefore, the modulation of aperiodic parameters and sample entropy after ketamine are perhaps indicative of this sustained cellular and functional normalization. Interestingly, our finding of an increased exponent in the mPFC after ketamine parallels findings after human electroconvulsive and magnetic seizure therapy (Smith et al., 2023). Furthermore, immediately after treatment, the GT also correlated with acutely decreased sample entropy and increased offset, while the FST correlated with increased offset and exponent. These findings indicate that, in addition to their potential as short- and long-term treatment efficacy biomarkers, these measures also have potential as immediate biomarkers. Similar to our interpretation of the sustained post-treatment electrophysiological correlates, we speculate that these immediate correlates represent processes critical to a rapid mPFC “normalization” mechanism. Rodent studies have found ketamine normalizes mPFC dopaminergic (Wu et al., 2021) and GABAergic signaling (Ghosal et al., 2020), the latter in particular potentially driving the observed normalization of the aperiodic parameters. Meanwhile human MRI signal in the medial frontal cortex is normalized via reductions in functional activity and connectivity immediately after ketamine during an emotionally-valenced stimulus task (Reed et al., 2018; Morris et al., 2020), potentially paralleling our finding of decreased sample entropy.

Interestingly, in addition to being highly correlated with remission-like behavior and one other at the same time points, sample entropy, offset, and the exponent were also correlated with one another across time points. Tracking these correlations across time may help bridge the gaps in understanding between the relatively well studied immediate correlates of ketamine’s efficacy, and the less well understood long-term correlates. Specifically, we found that the three measures correlated with an increase in the exponent at the short-term timepoint, which in turn correlated with decreased sample entropy immediately after treatment. In addition, we found that the long-term decrease in sample entropy correlated with a short-term decrease in the theta peak frequency, which in turn correlated with the immediate decrease in sample entropy, as well as the increase in the offset and low gamma peak width. Interestingly, unlike the other correlates, the shift in theta peak frequency was in the opposite direction of the control group, decreasing even more than in the CORT group. As previously discussed, modulation of peak theta frequency may relate to modulation of the hippocampal-mPFC pathway, which has previously been demonstrated after ketamine in humans (Siegel et al., 2021) and rats (Gass et al., 2019). Meanwhile, resting-state low gamma activity has been shown to increase in the mPFC after ketamine in both rodents (Qin et al., 2023) and humans (Cornwell et al., 2012), which correlated with treatment efficacy in humans (Nugent et al., 2019). Taken together, our findings potentially indicate that the increase of low gamma activity and inhibition, and decrease in complexity, immediately after ketamine treatment drove a short-term increase in inhibition and decrease in theta activity, which culminated in the remission-related long-term increase in inhibition and decrease in complexity. However, as this interpretation is based on correlations, we can not draw definitive conclusions regarding causality.

#### 4.1.3 The remission-like behavioral effects of mPFC DBS correlated with changes in the offset, low gamma parameters, sample entropy, and cognition-related behavior

After mPFC DBS alone, a long-term increase in the low gamma peak width and improvement in cognitive behavior in the NORT correlated with remission-like behavior in the GT. Furthermore, an increased offset at the short-term time point correlated with the FST. Finally, an immediate decrease in sample entropy, as well as the increase in the offset and low gamma peak width, all correlated with the GT. These correlations indicate these measures may serve as biomarkers for the long-term treatment efficacy after DBS, and when considering their neural origins and interrelationships, they may also hint at its mechanism of action. mPFC low gamma activity has previously been shown to increase to healthy levels after mPFC DBS in rodents (Jia et al., 2019) and humans (Scherer et al., 2023), and has been implicated in the cognitive functions of the mPFC, including memory in rodents (Zhang et al., 2019) and humans (Senkowski and Gallinat, 2015). Indeed, in the present study, improvements in object recognition memory in the NORT correlated with the immediate post-treatment increase in low gamma peak width. Furthermore, low gamma power has been shown to be generated via the interplay between glutamatergic and GABAergic neurons (Tort et al., 2013), and interestingly, cognitive deficits stemming from dysfunction of these cell types in the mPFC have been implicated in the etiology of apathy (Levy and Dubois, 2006). Particularly implicated are two components of recognition memory, working memory and episodic memory, which involve the mPFC (Ragozzino et al., 2002; Blumenfeld and Ranganath, 2007), and are measured by the NORT (Antunes and Biala, 2012). Furthermore, working memory performance has been previously correlated with apathy in human schizophrenia patients (Raffard et al., 2016). Therefore, our findings hint that immediate and long-term modulation of glutamatergic and GABAergic neuron interplay may have driven long-term improvements in cognition that then related to remission-like performance in the GT. The translatability of this finding is supported by work in humans showing that mPFC DBS increases gamma activity (Scherer et al., 2023) and improves long-term measures of memory (Runia et al., 2023). Again, we must be cautious in overinterpreting these correlations, more work will need to be done to demonstrate causal links, if any. Furthermore, it is unclear how the immediate and short-term modulation of complexity and inhibition could causally relate to the long-term depression-related behavioral measures given the lack of intermediate correlates and relatively weak modulation, in comparison to ketamine treatment.

#### 4.1.4 The apathy-like behavior of the combinatorial group only correlated with a failure to suppress an increase in sample entropy immediately after treatment

Finally, we hypothesized that a novel combinatorial treatment of ketamine and mPFC DBS would be synergistically effective in its antidepressant-like effect. As in both treatments separately, this strategy rescued despair-like symptoms in the FST, however, it failed to rescue apathy-like symptoms in the GT (Figure 2). To search for an explanation for these counterintuitive findings, we looked for immediate, short- and long-term mPFC LFP correlates of the GT. The only hint we uncovered was a negative correlation with the increase in sample entropy immediately after treatment, on par with the CORT group, and in contrast to the control and separate treatment groups. Across the aforementioned biomarkers of the GT and FST, the combination group resembled a mixture of the other groups, which, coupled with a lack of correlations at the short- and long-term time points, hinders the identification of a potential mechanism across time. However, by investigating this interference in the modulation of mPFC activity further, future studies may uncover causal relationships between electrophysiological and behavioral changes.

### 4.2 Limitations

There are several important limitations to the present study. Sex-based differences were not explored here, though there is evidence of sex-differences in human patients and rodent models of depression (Breslau et al., 1995; Bertholomey et al., 2022). Also, the animals were single housed, which may have altered behavior (Liu et al., 2020). Furthermore, the duration of time immediately post-treatment, 20 min, that was studied here may not have captured all therapeutically-relevant electrophysiological changes induced by the treatments, particularly ketamine (Caixeta et al., 2013). Moreover, without groups that received KET and/or DBS with no CORT, we cannot compare the electrophysiological effects of these treatments in healthy versus stressed brains, limiting the possible interpretations of our data. Finally, due to faulty equipment, weekly SPTs were excluded from the study.

### 4.3 Conclusion

This study contributes to the growing evidence that electrophysiological measures of the mPFC correlate with behavioral symptoms of depression, as well as the antidepressant action of DBS and ketamine. Therefore, it supports their potential as predictive biomarkers for depression severity and treatment efficacy. Specifically, our study supports previous clinical studies that correlate the exponent and increased inhibition with depression, and is among the first preclinical studies to find a direct correlation between aperiodic-adjusted theta parameters, sample entropy, aperiodic parameters, and depression-like symptoms. For ketamine treatment, our findings support previous studies showing immediate and sustained normalization of mPFC function after ketamine treatment, and novelly demonstrates a correlation between normalization of LFP aperiodic parameters and sample entropy with remission-like behavior after ketamine. This study also supports previous studies demonstrating low gamma parameter modulation and cognitive improvements after mPFC DBS, and is among the first to correlate these measures, as well as sample entropy and aperiodic offset, with remission-like behavior. Finally, this study provides strong evidence that the offset, exponent, and especially sample entropy are potentially universal, cross-modal predictive biomarkers for depression, post-treatment remission, and failed treatment. Further exploration of the mechanisms that interconnect electrophysiological and behavior changes will enable the development of biomarkers for targeted, personalized, and monitored treatment strategies that can address the weaknesses of current treatments. Finally, the complex interaction between the two treatments studied here enhance our understanding of their separate mechanisms and demonstrate that the combination of these treatments may be detrimental to certain aspects of therapeutic efficacy. This needs to be explored further, but the nature of their interaction may be critical information for patients and clinicians.

## Data availability statement

The raw data supporting the conclusions of this article will be made available by the authors, without undue reservation.

## Ethics statement

The animal study was approved by USC Institutional Animal Care and Use Committee. The study was conducted in accordance with the local legislation and institutional requirements.

## Author contributions

MB: Conceptualization, Data curation, Formal analysis, Investigation, Methodology, Project administration, Supervision, Visualization, Writing – original draft, Writing – review & editing. SM: Conceptualization, Investigation, Methodology, Supervision, Writing – review & editing. NZ: Conceptualization, Methodology, Project administration, Supervision, Writing – review & editing. LC: Conceptualization, Investigation, Methodology, Software, Supervision, Visualization, Writing – review & editing. JI: Investigation, Writing – review & editing. LD: Investigation, Writing – review & editing. LR: Investigation, Writing – review & editing. EA: Investigation, Writing – review & editing. TL: Investigation, Writing – review & editing. EH: Investigation, Writing – review & editing. WC: Investigation, Writing – review & editing. KW: Investigation, Writing – review & editing. JL: Investigation, Writing – review & editing. DL: Conceptualization, Project administration, Resources, Supervision, Writing – review & editing.
