# Supplementary material for: Immediate and long-term electrophysiological biomarkers of antidepressant-like behavioral effects after subanesthetic ketamine and medial prefrontal cortex deep brain stimulation treatment
Source: Front Neurosci. 2024 Jun 20;18:1389096. doi: 10.3389/fnins.2024.1389096 (PMC11222339; doi:10.3389/fnins.2024.1389096)

**1. Supplemental Methods**

Novel Object Recognition Test

On PSD 31, the animals were habituated to the test arena during the OFT for 10 minutes. On PSD 32, they explored the arena which contained two identical objects: Object A, a black Monster Energy can (Monster Energy Company, Corona, CA), in the position it would occupy in all trials; and Object A’, in the position the novel objects would occupy. On PSD 33 the arena contained Object A; and a novel object, Object B, a white Monster Energy can. On PSD 34 the arena contained Object A; and a new novel object, Object C, a Powerade bottle (The Coca-Cola Company, Atlanta, GA). Video recordings were analyzed of each 5 minute trial on PSD 32-34 to quantify the number of seconds spent by the animal exploring each object. The Discrimination Index (DI) of each trial was calculated by subtracting the time each animal spent exploring the familiar object (A) from the time spent with the novel object (A’, B, C), and dividing by the total time exploring both objects. DI results of both test days (PSD 33 and 34) of an animal were averaged, then the DI of the familiarization day (PSD 32) was subtracted. This difference was normalized by dividing it by the familiarization day DI and multiplying by 100 to generate a percent change that was averaged across animals in a group.

Elevated Plus Maze

The EPM is a plus-shaped maze, with two opposing “closed” arms with sidewalls (40 cm tall), and two opposing “open” arms with no sidewalls. The lanes created by two opposing arms are 110 cm long, 10 cm wide, and 62 cm high. The animals were placed at the center of the maze and allowed to explore for 5 minutes. Video recordings were analyzed for the number of seconds spent in the open arms, which was divided by the total seconds of the trial, to calculate the percent of time spent in the open arms.

**2. Supplemental Results**

Table S1. Detailed results of behavioral post-hoc tests. One-sample tests were used to compare a group’s percent change in a measurement to its own baseline (percent change/mu = 0), while two-sample tests were done when comparing between groups. Assumptions were checked so that appropriate tests were done, as described in the Methods. (PSD = Post Surgery Day, CORT = Corticosterone, KET = Ketamine, DBS = Deep Brain Stimulation, FDR = False Discovery Rate)

| Row | Measure | Control | CORT | CORT+KET | CORT+DBS | CORT+DBS+KET |
| --- | --- | --- | --- | --- | --- | --- |
| 1 | Groom Test  Corrected | n = 8  CORT:  F(7,7) = 1.45,  p = .032), | n = 8 | n = 9  CORT:  F(8,7) = .40,  p = .032 | n = 8  CORT:  F(7,7) = .27,  p = .032 | n = 9 |
| 2 | Forced Swim Test  Corrected | CORT:  F(7,7) = .39,  p = .012 |  | CORT:  Z(8,7) = -2.60,  p = .022 | CORT:  F(7,7) = 2.45,  p = .022 | CORT:  Z = -2.46,  p = .022 |
| 3 | Novel Object Recognition Test  Corrected |  |  |  | CORT:  Z(7,7) = -3.26,  p < .01 |  |
| 4 | Locomotion  Corrected |  |  |  |  | Control:  F(8,7) = .54,  p = .036 |
| 5 | PSD 31 Relative Weight  Corrected |  | Control:  F(7,7) = 0.84,  p < .0001 | Control:  F(8,7) = .49,  p < .0001 | Control:  F(7,7) = 0.40,  p < .001 | Control:  F(8,7) = .52,  p < .0001 |
| 6 | PSD 38  Relative Weight  Corrected |  | Control:  F(7,7) = 0.56,  p = .015 | Control:  F(8,7) = .49,  p < .01 | Control:  F(7,7) = 0.48,  p < .01 | Control:  F(8,7) = .40,  p < .01 |

Figure S1. Changes in sample entropy immediately after treatment, PSD 24+29, across the recommended parameters for *r* and *m*. Sample entropy measures the negative natural logarithm of the conditional probability that a sequence that matches for *m* points will continue to match at the next point, within a tolerance *r*. Higher sample entropy is therefore interpreted as higher irregularity or complexity, as the probability is lower of finding a match. Across all parameter combinations tested, sample entropy in the mPFC of the CORT group significantly increased relative to its pretreatment baseline on PSD 24+29 (indicated by ‘*’), while decreasing in the control and increasing in the CORT+DBS+KET groups in the majority of pairings. Furthermore, the CORT group significantly increased relative to the control group in every pairing (indicated by ‘#’), and in all but one pairing in the CORT+KET group. (*, #: p < .05 in FDR-corrected one/two sample tests; **, ##: p < .01; CORT = Corticosterone, PSD = Post Surgery Day, DBS = Deep Brain Stimulation, KET = Ketamine, FDR = False Discovery Rate)


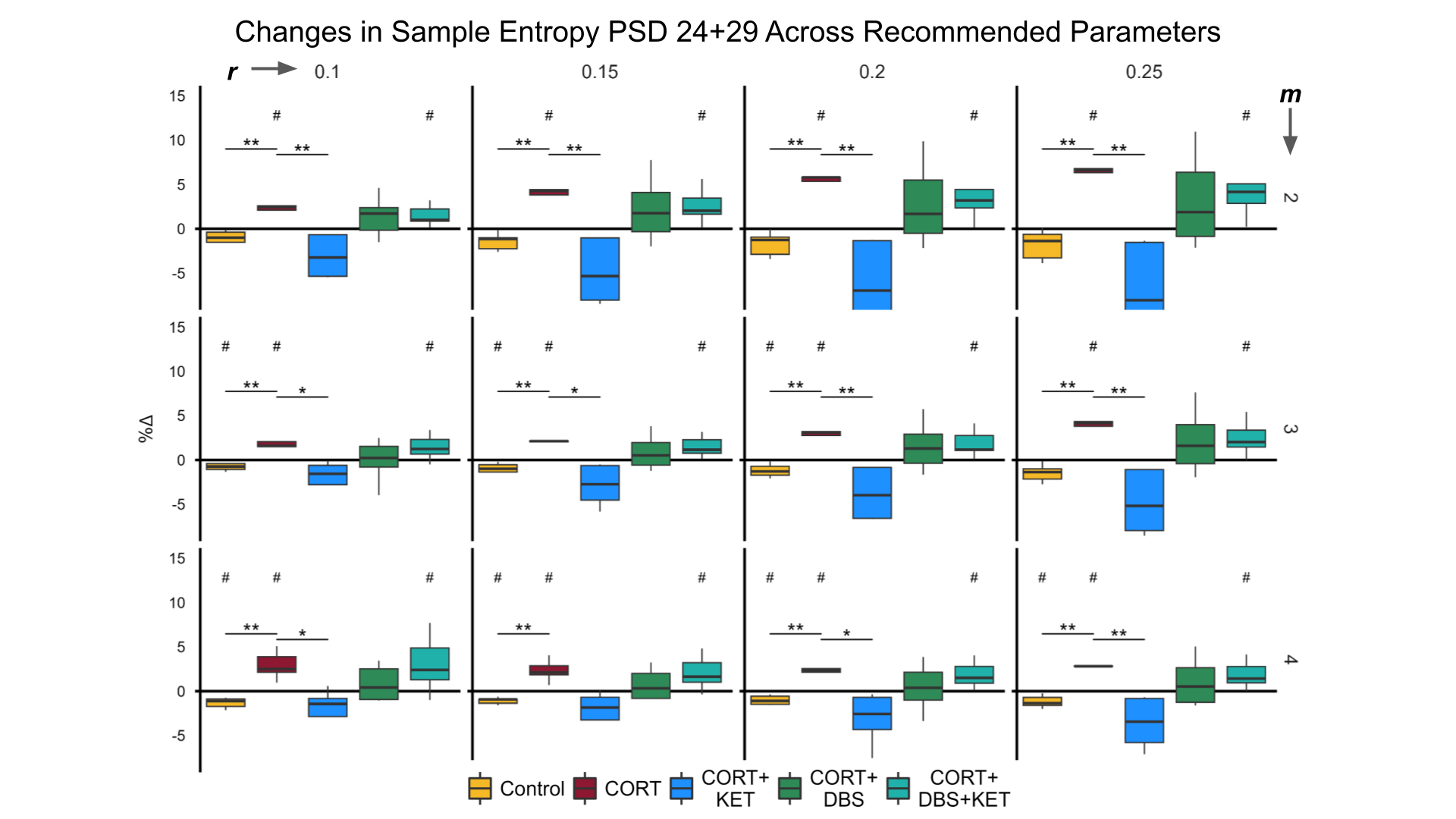


Figure S2. Changes in low gamma spectral parameters. Boxplots of the percent change in periodic low gamma (20-50 Hz) spectral parameters: power (mV^2^/Hz), peak frequency (Hz), and peak width (Hz). (A) On PSD 24+29, peak frequency in the control group increased significantly relative to baseline (indicated by ‘#’), and trendwise relative to the CORT group (indicated by ‘*’). Peak width was decreased trendwise in the CORT group relative to the CORT+DBS and CORT+KET groups. (B) On PSD 31, low gamma power was significantly increased in the CORT+KET+DBS group. (C) Power, peak frequency, and peak width were decreased in the CORT group, and peak frequency was decreased trendwise in the CORT+KET group. (*, #: p < .05 in uncorrected one/two sample tests; **, ##: p < .01; CORT = Corticosterone, PSD = Post Surgery Day, DBS = Deep Brain Stimulation, KET = Ketamine, Freq = Peak Frequency)


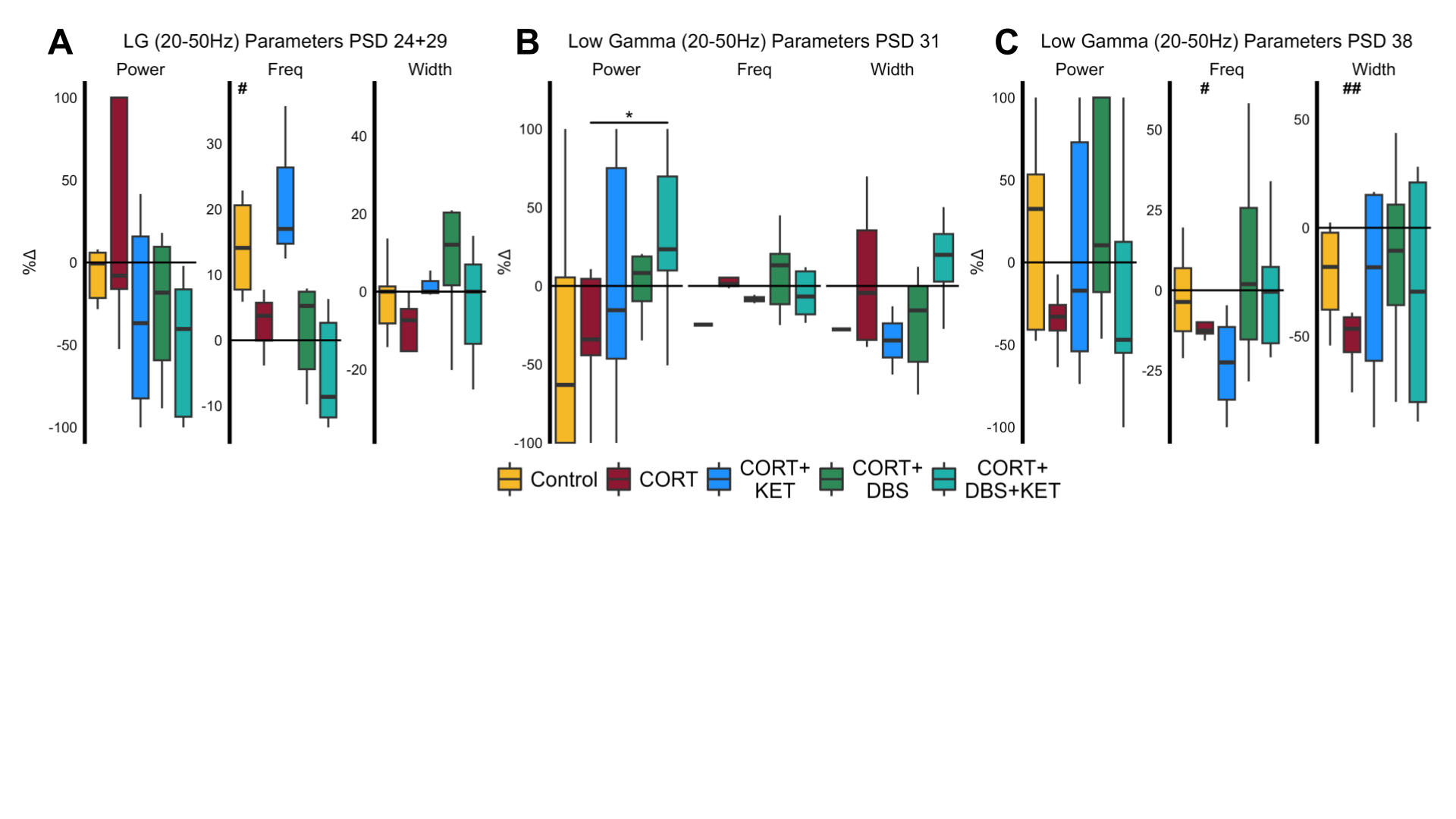


Table S2. Detailed results of electrophysiological post-hoc tests. One-sample tests were used to compare a group’s percent change in a measurement to its own baseline (percent change/mu = 0), while two-sample tests were done between a group and the CORT group. Assumptions were checked so that appropriate tests were done, as described in the Methods. (PSD = Post Surgery Day, CORT = Corticosterone, KET = Ketamine, DBS = Deep Brain Stimulation, FDR = False Discovery Rate)

| Row | Measure | Control | CORT | CORT+KET | CORT+DBS | CORT+DBS+KET |
| --- | --- | --- | --- | --- | --- | --- |
| 1 | Periodic Spectral Parameters PSD 24+29  Uncorrected | Baseline:  Theta Peak Frequency  t(4) = 3.05,  p = .038  Low Gamma Peak Frequency  t(3) = 3.40,  p = .042  CORT:  Theta Power  F(4,4) = 1.48,  p < .01  Theta Peak Frequency  F(4,4) = 1.07,  p = .074  Low Gamma Peak Frequency  F(3,2) = .23,  p = .082 | Baseline:  Theta Power  t(4) = -3.55,  p = .024 | Baseline:  Theta Power  t(4) = -6.95,  p < .01  CORT:  Low Gamma Peak Width  Z(7,3) = -1.73,  p = .084 | Baseline:  Theta Power  t(6) = -3.56,  p = .012  CORT:  Low Gamma Peak Width  t(6,3) = 2.10,  p = .062 | CORT:  Theta Power  Z(5,4) = 2.62,  p < .01 |
| 2 | Aperiodic Spectral Parameters PSD 24+29  Uncorrected | CORT:  Exponent  F(4,4) = 3.24,  p = .60 | Baseline:  Offset  t(4) = -3.32,  p = .029 | Baseline:  Exponent  t(4) =3.69,  p = .021  Offset  t(4) = 3.41,  p = .027  CORT:  Offset  F(4,4) = .13,  p < .01  Exponent  F(4,4) = .57,  p < .01 |  | Baseline:  Exponent  t(8) = -2.92,  p = .019  Offset  t(7) = -2.79,  p = .027  CORT:  Exponent  F(8,4) = 7.16,  p = .060 |
| 3 | Sample Entropy PSD 24+29  FDR- Corrected | Baseline:  t(4) = -3.72,  p = .034  CORT:  F(4,3) = .25,  p < .01 | Baseline:  t(4) = -4.62,  p = .034 | Baseline:  t(5) = -2.75,  p = .064  CORT:  F(4,4) = .24,  p < .01 |  | Baseline:  t(8) = 3.07,  p = .034 |
| 4 | Periodic Spectral Parameters PSD 31  Uncorrected |  |  | CORT:  Theta Peak Frequency  Z(5,4) = -2.17  p = .030 |  | CORT:  Low Gamma Power  F(6,4) = 1.44  p = .044 |
| 5 | Aperiodic Spectral Parameters PSD 31  Uncorrected | CORT:  Exponent  F(4,3) = 2.86,  p = .081 |  |  | Baseline:  Offset  t(5) = -4.05,  p < .01  Exponent  t(5) = -4.14,  p < .01 |  |
| 6 | Periodic Spectral Parameters PSD 38  Uncorrected | Baseline:  Theta Peak Frequency  t(4) = -3.46,  p = .026 | Baseline:  Theta Power  t(3) = 7.48,  p < .01  Theta Peak Frequency  t(3) = -3.24,  p = .048  Low Gamma Peak Bandwidth  t(3) = -6.23,  p < .01  Low Gamma Peak Frequency  t(3) = -3.94,  p = .029  Low Gamma Power  F(3) = -2.97,  p = .059 | Baseline:  Theta Power  t(5) = -4.14,  p < .01  Theta Peak Frequency  t(5) = -4.47,  p < .01  Low Gamma Peak Frequency  t(3) = -2.70,  p = .074  CORT:  Theta Power  F(5,3) = .03,  p = .053  Theta Peak Frequency  F(5,3) = .21,  p = .053 |  | Baseline:  Theta Power  t(6) = -2.52,  p = .045 |
| 7 | Aperiodic Spectral Parameters PSD 38  Uncorrected | CORT:  Exponent  F(3,3) = 4.90,  p = .060 | Baseline:  Exponent  t(3) = -3.47,  p = .040  Offset  Z(4) = -1.86,  p = .063 | Baseline:  Exponent  t(4) = 2.57,  p = .062  CORT:  Exponent  F(4,3) = .76,  p < .01  Offset  Z(4,2) = -2.41,  p = .012 | Baseline:  Exponent  t(4) = -5.58,  p < .01 | CORT:  Exponent  Z(6,3) = -1.79,  p = .073  Offset  Z(6,4) = -1.79,  p = .073 |
| 8 | Sample Entropy PSD 38  FDR- Corrected | CORT:  F(4,3) = 25.0,  p = .024 | Baseline:  t(3) = 10.89,  p < .01 | Baseline:  Z(5) = -2.15,  p = .078  CORT:  Z(5,3) = -2.59,  p = .024 | CORT:  Z(4,3) = -2.14,  p = .042 |  |

Figure S3. Changes in spectral parameters one day post-treatment, PSD 31. (A) Median aperiodic-adjusted periodic power spectrum density plots of the pre-CORT PSD 10 baseline local field potential recordings (black), and the short-term post-treatment PSD 31 recording (colored) for each group. (B) Boxplots of the percent change in periodic theta (5-10 Hz) spectral parameters: power (mV2/Hz), peak frequency (Hz), and peak width (Hz). The peak frequency of theta significantly decreased in the CORT and CORT+KET group compared to their baseline (indicated by ‘#’), and it decreased more in the CORT+KET group than the CORT group (indicated by ‘*’). (C) Boxplots of the percent change in aperiodic spectral parameters, offset and exponent. The offset in the CORT group decreased significantly from baseline, and trendwise from the control group. Both the offset and the exponent decreased significantly in the CORT+DBS group. (*, #: p < .05 in uncorrected one/two sample tests; **, ##: p < .01; CORT = Corticosterone, PSD = Post Surgery Day, DBS = Deep Brain Stimulation, KET = Ketamine, Freq = Peak Frequency)


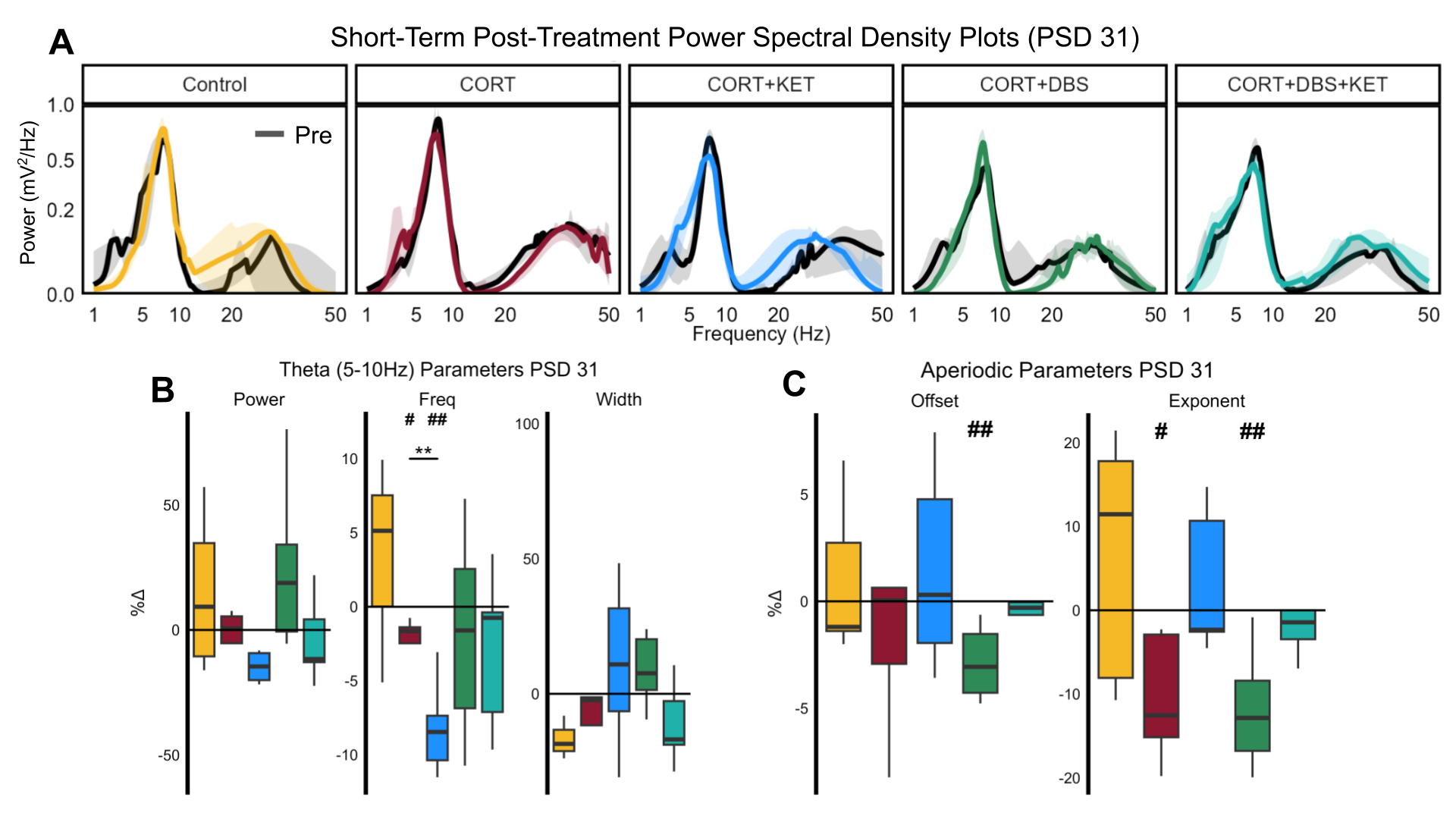


Figure S4. Changes in sample entropy one week after treatment, PSD 38, across the recommended parameters for *r* and *m*. Sample entropy significantly increased in the CORT group relative to the control and CORT+KET groups in all 12 parameter combinations (indicated by ‘*’), but only one relative to the CORT+DBS group (m = 4, r = .25). The CORT group increased significantly from pre-CORT baseline on PSD 10 in 7/12 parameter combinations tested (indicated by ‘#’). (*, #: p < .05 in FDR-corrected one/two sample tests; **, ##: p < .01; CORT = Corticosterone, PSD = Post Surgery Day, DBS = Deep Brain Stimulation, KET = Ketamine, FDR = False Discovery Rate)


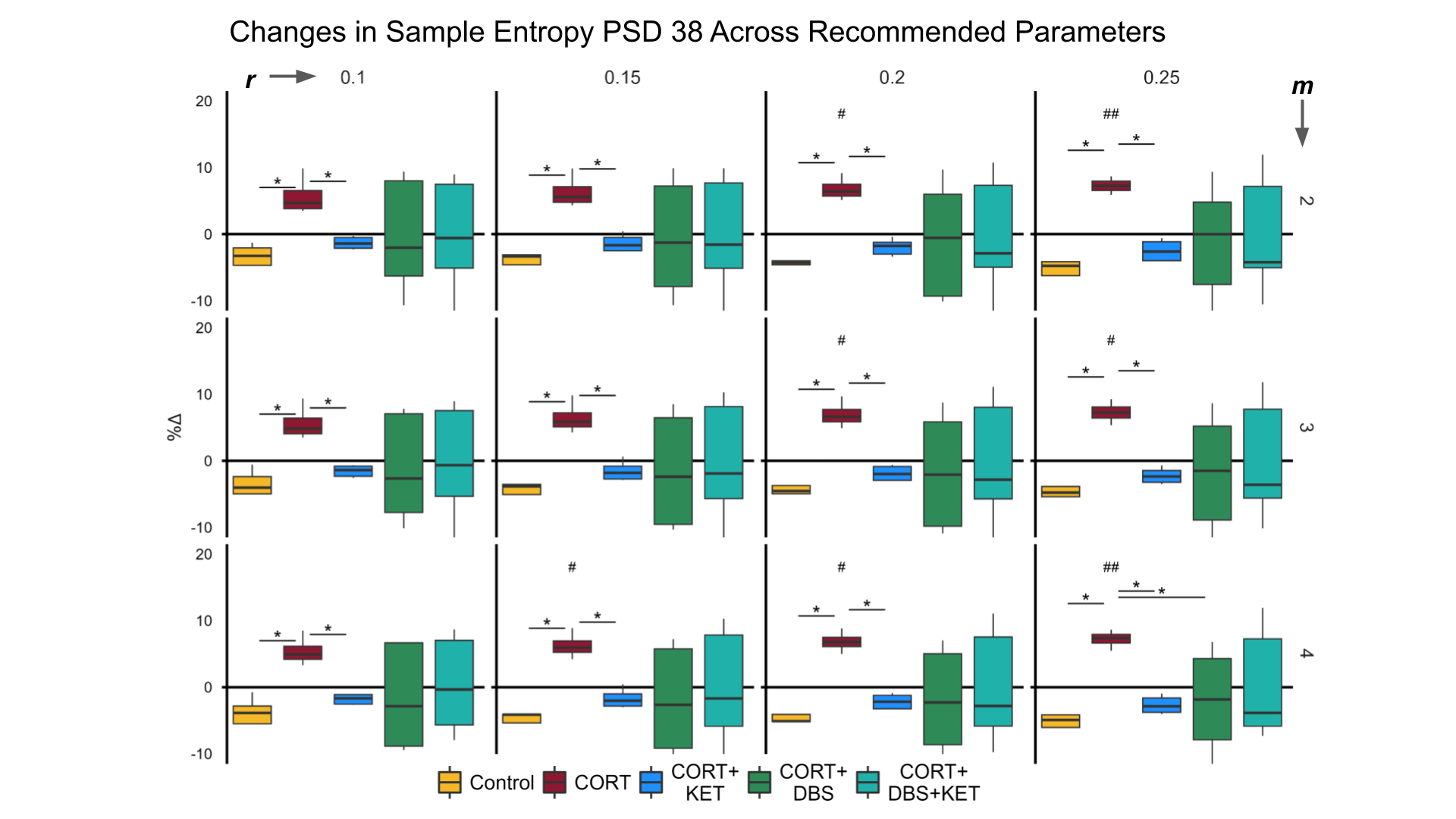


Table S3. Correlation tests and results. The coefficients and p-values of nine sets of linear Pearson or Spearman correlations between electrophysiological and behavioral measures were calculated. Each group’s measure results were pooled with those of the CORT group to create “group pools”. All tests were uncorrected, and assumptions were checked so the appropriate type of correlation was applied, as described in the methods. (PSD = Post Surgery Day, CORT = Corticosterone, GT = Groom Test, FST = Forced Swim Test, NORT = Novel Object Recognition Test, OFT = Open Field Test, SE = Sample Entropy, Ephys = Electrophysiology)

| Row | Relationship | Control x CORT Group Pool | CORT x CORT +KET Group Pool | CORT x CORT+DBS Group Pool | CORT x CORT+KET+  DBS Group Pool |
| --- | --- | --- | --- | --- | --- |
| 1 | GT/FST x  PSD 38 Ephys | GT x Theta Peak Frequency:  r = -.94,  p < .001 | GT x Exponent:  r = .91,  p < .001  GT x Offset:  r = .91,  p < .001  FST x Offset:  r = -.79,  p < .01  GT x SE:  r = -.67,  p = .033 | GT x Low Gamma Peak Width:  r = .71,  p = .047 |  |
| 2 | GT/FST x NORT/PSD 31 Ephys | GT x Exponent:  r = .88,  p < .001 | GT x Exponent:  r = .83,  p < .01 | FST x Offset:  r = .64,  p = .035  GT x NORT:  r = .56,  p = .019 |  |
| 3 | GT/FST x  PSD 24+29 Ephys | GT x SE: r = -.75,  p = .013  GT x Theta Power: r = .63,  p = .051 | GT x SE: r = -.72,  p = .020  GT x Offset: r = .78,  p < .01  FST x Exponent: r = -.75,  p = .013  FST x Offset: r = -.75,  p = .013 | GT x SE: r = -.65,  p = .031  GT x Low Gamma Peak Width: r = .66,  p = .026  GT x Offset: r = .68,  p = .015 | GT x SE: rho = -.61,  p = .020 |
| 4 | PSD 38 x  PSD 31 Ephys | Theta Peak Frequency x Exponent: r = -.94,  p < .001 | Exponent x Exponent:  r = .79,  p = .024  Offset x Exponent:  rho = .75,  p = .013  SE x Exponent  r = -.73,  p = .026  SE x Theta Peak Frequency:  r = .69,  p = .040 |  |  |
| 5 | PSD 31 x PSD 24+29 Ephys | Exponent x Theta Power:  r = .71,  p = .028 | Exponent x SE:  r = -.74,  p = .037  Theta Peak Frequency x SE:  r = .84,  p < .01  Theta Peak Frequency x Offset:  r = -.75,  p = .029  Theta Peak Frequency x Low Gamma Peak Width:  r = -.94,  p = .016 | NORT x Low Gamma Peak Width:  r = .71,  p = .013 |  |
| 6 | Weight x Ephys | Weight PSD 38 x Exponent PSD 38:  r = .74, p = .034  Exponent PSD 38 x Exponent PSD 31:  r = .69, p = .057  Weight PSD 31 x Exponent PSD 24+29:  r = .67, p = .034  Weight PSD 31 x Offset PSD 24+29:  r = .65, p = .043  Weight PSD 31 x SE PSD 24+29:  r = -.71, p = .031  Weight PSD 31 x Theta Peak Frequency PSD 24+29:  r = .70, p = .025 |  |  |  |
| 7 | GT/FST x Weight PSD 38/OFT Average Velocity | GT x OFT Average Velocity:  r = .51, p = .043 |  |  |  |
| 8 | SE PSD 24+29 x Spectral Parameters PSD 24+29 | SE x Theta Power:  r = -.77, p = .018 | SE x Offset:  r = -.94, p < .0001  SE x Exponent:  r = -.89, p < .0001 | SE x Exponent:  r = -.62, p = .040 |  |
| 9 | SE PSD 38 x Spectral Parameters PSD 38 | SE x Low Gamma Peak Width:  r = -.75, p = .033  SE x Exponent:  r = -.83, p = .020 | SE x Exponent:  r = -.83, p = .010  SE x Offset:  r = -.82, p < .01 |  | SE x Exponent:  r = -.87, p < .01  SE x Offset:  r = -.72, p = .014  SE x Theta Power :  r = -.62, p = .044 |

Figure S5. Electrophysiological and behavioral measures correlated with one another across time. (A-H) Long-term post-treatment electrophysiological measures (PSD 38) that correlated with the GT or FST were correlated with short-term post-treatment (PSD 31) measures. Short-term measures that significantly correlated were then correlated with immediate post-treatment (PSD 24+29) measures. (PSD = Post Surgery Day, GT = Groom Test, FST = Forced Swim Test, SampEn/SE = Sample Entropy, CORT = Corticosterone, KET = Ketamine, DBS = Deep Brain Stimulation, Exp = Exponent, Freq = Peak Frequency, Pow = Power, NORT = Novel Object Recognition Test, LG = Low Gamma)


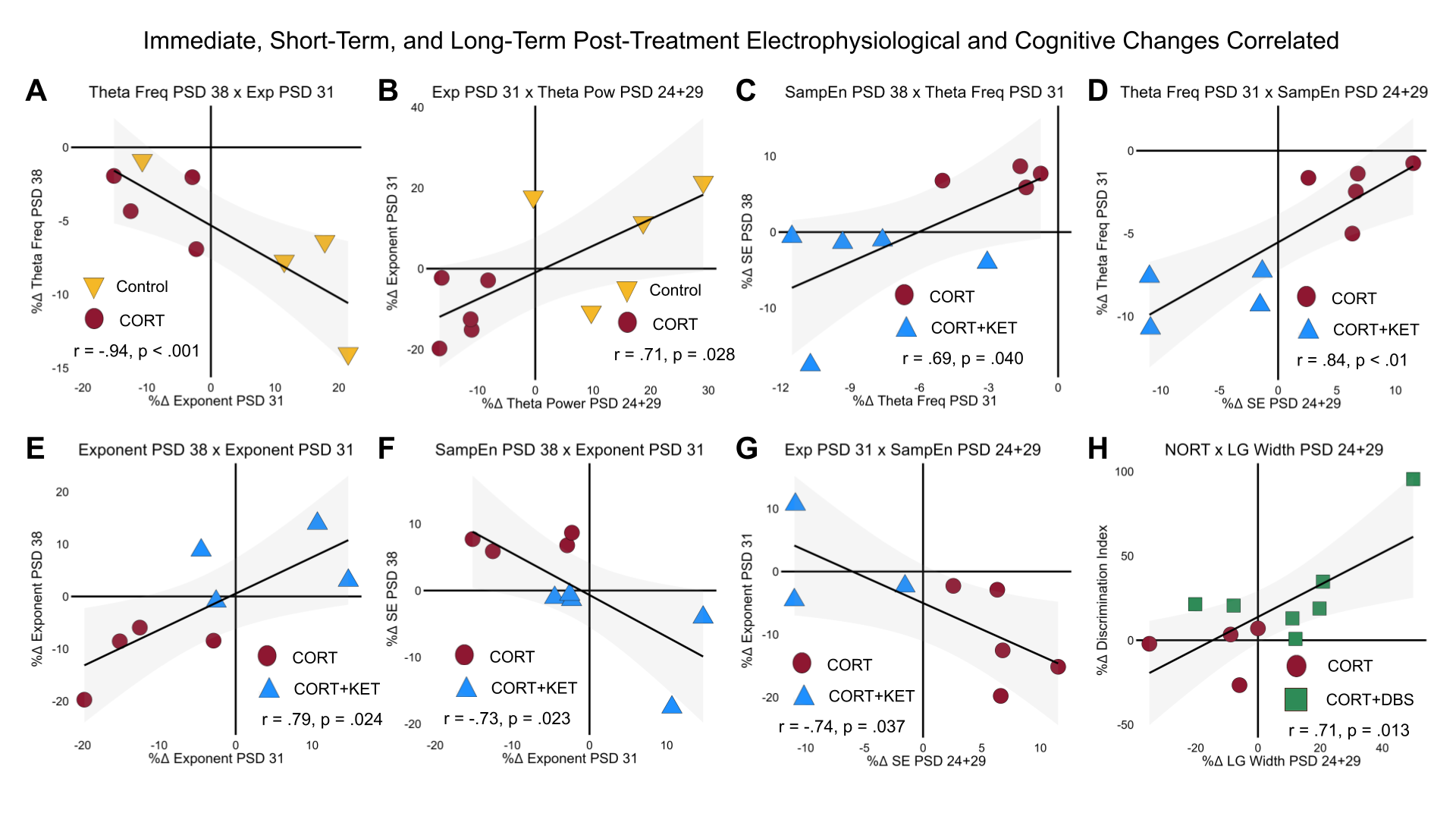


Figure S6. Electrophysiological correlates of weight and sample entropy. (A) Relative weight at the long-term post-CORT time point, PSD 38, significantly correlated with the exponent on PSD 31, (B) which in turn correlated trendwise with the exponent on PSD 31. Relative weight on PSD 31 was correlated with the (C) exponent and (D) sample entropy immediately after sham-treatment. As the neural underpinnings of sample entropy are not well understood, we tested whether sample entropy significantly correlated with spectral parameters. (E-F) Sample entropy consistently correlated with the exponent, and at times with theta power and low gamma peak width. (PSD = Post Surgery Day, CORT = Corticosterone, SampEn/SE = Sample Entropy, Exp = Exponent, LG = Low Gamma)


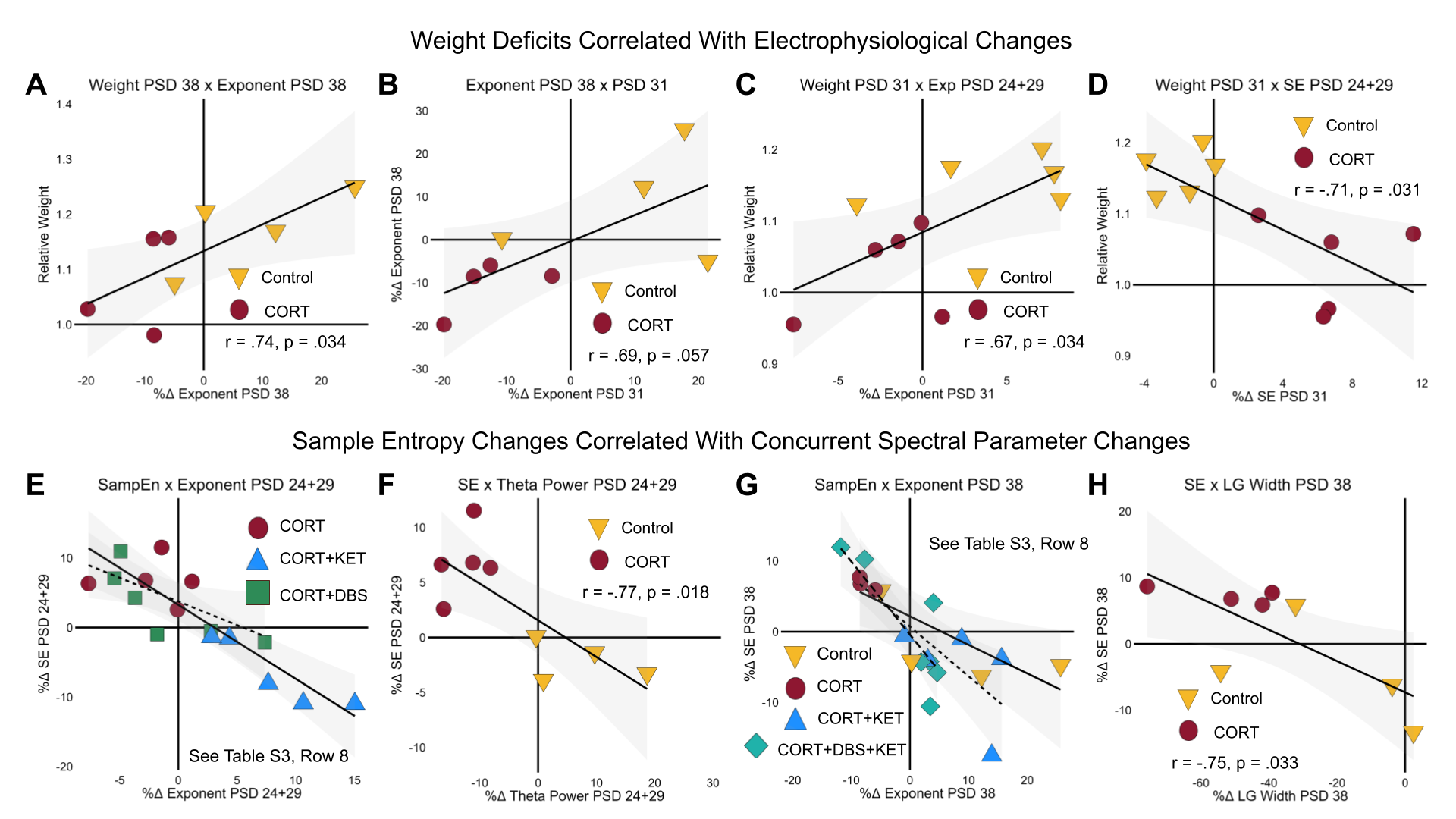

Supplement: Supplementary file 1 [file Data_Sheet_1.docx]
